# Determinants of suicidal ideation and suicide attempts: parallel cross-sectional analyses examining geographical location

**DOI:** 10.1186/1471-244X-14-208

**Published:** 2014-07-23

**Authors:** Kerry J Inder, Tonelle E Handley, Amy Johnston, Natasha Weaver, Clare Coleman, Terry J Lewin, Tim Slade, Brian J Kelly

**Affiliations:** Centre for Translational Neuroscience and Mental Health, University of Newcastle, Callaghan, Newcastle, New South Wales (NSW) Australia; Hunter Medical Research Institute, Newcastle, NSW Australia; Centre for Rural and Remote Mental Health, University of Newcastle, Newcastle, NSW Australia; National Drug and Alcohol Research Centre, University of New South Wales, Sydney, NSW Australia; School of Medicine and Public Health, University of Newcastle, Callaghan, Newcastle, NSW Australia; Sydney Centre for Aboriginal and Torres Strait Islander Statistics, Faculty of Health Sciences, University of Sydney, Sydney, Australia; Hunter New England Mental Health Service, Newcastle, NSW Australia

**Keywords:** Rural mental health, Suicidal ideation, Suicide attempts, Psychiatric disorder, Remoteness, Social determinants of health

## Abstract

**Background:**

Suicide death rates in Australia are higher in rural than urban communities however the contributors to this difference remain unclear. Geographical differences in suicidal ideation and attempts were explored using two datasets encompassing urban and rural community residents to examine associations between socioeconomic, demographic and mental health factors. Differing patterns of association between psychiatric disorder and suicidal ideation and attempts as geographical remoteness increased were investigated.

**Methods:**

Parallel cross-sectional analyses were undertaken using data from the 2007 National Survey of Mental Health and Wellbeing (2007-NSMHWB, n = 8,463), under-representative of remote and very remote residents, and selected participants from the Australian Rural Mental Health Study (ARMHS, n = 634), over-representative of remote and very remote residents. Uniform measures of suicidal ideation and attempts and mental disorder using the World Mental Health Composite International Diagnostic Interview (WMH-CIDI-3.0) were used in both datasets. Geographic region was classified into major cities, inner regional and other. A series of logistic regressions were undertaken for the outcomes of 12-month and lifetime suicidal ideation and lifetime suicide attempts, adjusting for age, gender and psychological distress. A sub-analysis of the ARMHS sample was undertaken with additional variables not available in the 2007-NSMHWB dataset.

**Results:**

Rates and determinants of suicidal ideation and suicide attempts across geographical region were similar. Psychiatric disorder was the main determinant of 12-month and lifetime suicidal ideation and lifetime suicide attempts across all geographical regions. For lifetime suicidal ideation and attempts, marital status, employment status, perceived financial adversity and mental health service use were also important determinants. In the ARMHS sub-analysis, higher optimism and better perceived infrastructure and service accessibility tended to be associated with a lower likelihood of lifetime suicidal ideation, when age, gender, psychological distress, marital status and mental health service use were taken into account.

**Conclusions:**

Rates and determinants of suicidal ideation and attempts did not differ according to geographical location. Psychiatric disorder, current distress, employment and financial adversity remain important factors associated with suicidal ideation and attempts across all regions in Australia. Regional characteristics that influence availability of services and lower personal optimism may also be associated with suicidal ideation in rural communities.

**Electronic supplementary material:**

The online version of this article (doi:10.1186/1471-244X-14-208) contains supplementary material, which is available to authorized users.

## Background

Suicide death rates have been consistently higher in rural than in urban settings across all states and territories of Australia between 2001 and 2010 [1], particularly in men [2]. Psychiatric disorder, particularly depression, is the strongest predictor of suicide death rates, [3] however the prevalence of depression is similar across urban and rural areas in Australia [4] and elsewhere [5]. Differences in urban and rural suicide rates have been linked to a number of factors other than mental health including socio-demographic factors [6], health service related factors such as access and availability of services [7, 8], and access to lethal means [9].

People who experience suicidal ideation and those who make suicide plans are at increased risk of suicide attempts, and people who experience all forms of suicidal thoughts and behaviours are at greater risk of completed suicide [4]. An estimated 13.3% of community dwelling adults in Australia experience suicidal ideation during their lifetime with 3.2% attempting suicide during their lifetime, highlighting an important public health problem [10]. Suicide deaths are higher in males; however suicidal ideation and behaviours occur more commonly among females, younger people, those outside the workforce, and those experiencing a mental disorder [10].

While the 2007 Australian National Survey of Mental Health and Wellbeing (NSMHWB) was under-representative of residents from remote and very remote communities [11], recent suicidality research in an exclusively rural sample obtained largely similar results [12]. In a rural population in New South Wales, Australia, suicidal ideation was predicted by younger age, being currently unmarried, not being in the workforce, and lifetime anxiety and substance use disorders, after controlling for depression, the strongest predictor of suicidal ideation [12]. This indicates the importance of the presence of mental disorder among people with suicidal ideation. This also suggests that rural/urban differences in suicide may be best examined by investigating differing triggers from the presence of disorder to the development of suicidal ideation and behaviour. These triggers may involve differing social contexts (e.g., potential mediators such as social support and service availability), differing levels of severity of symptoms (e.g., through delays in presentation for treatment) or differing patterns of psychiatric comorbidity.

The relationship between suicidal behaviour and geographical location has previously been explored [13, 14], focusing on the extent of the difference between urban and rural suicide rates [15]. A major limitation of existing studies exploring the relationship between suicidal behaviour and geographical location is that they conceptualise location as a dichotomous construct, exploring ‘urban’ versus ‘rural’ areas, and frequently under-represent remote and very remote residents [16]. Such an approach may obscure differences between ‘rural’ communities, which may be highly heterogeneous, restricting our understanding of the potential influence of residence on mental health [17]. Exploring this issue using a broader classification of locality may enhance our ability to identify differential effects between geographical locations and in particular, may reveal factors that contribute specifically to rural suicide [17].

This study draws upon two datasets with uniform measures of suicidal ideation, suicide attempts and mental disorder to examine associations between demographic, socioeconomic, mental and physical health factors and suicidal ideation and suicide attempts across urban, regional, and rural/remote regions. We investigated differing patterns of association between psychiatric disorder and suicidal ideation and suicide attempts as geographical remoteness increased, hypothesising that 12-month and lifetime rates of suicidal ideation and lifetime suicide attempts would be significantly higher in more remote residents.

## Methods

### Sampling strategies

Data were obtained from two existing complimentary studies (the 2007-NSMHWB and the Australian Rural Mental Health Study, ARMHS) to undertake parallel cross-sectional analyses to explore the determinants of suicidal ideation and suicide attempts across geographical regions. Sampling for both studies involved randomly selected participants residing in private dwellings and both studies used the same standardised and validated instrument to measure suicidal ideation and suicide attempts at a similar point in time.

The 2007-NSMHWB, conducted between August and December 2007 by the Australian Bureau of Statistics (ABS), assessed the prevalence of mental disorders in Australia using the World Mental Health Composite International Diagnostic Interview version three (WMH-CIDI-3.0) [18], administered by face-to-face interviews. Following informed consent, a total of 8,463 Australian residents aged 18 to 85 years completed the interview (response rate 60%); however, respondents were under-representative of remote and very remote residents [11].

The ARMHS is a longitudinal cohort study that aims to examine the determinants of mental health in rural and remote communities, including suicidal ideation and attempts [19]. Of the 2,638 non-metropolitan participants aged 18 years and over who gave informed consent and completed the ARMHS baseline postal survey (response rate 27%) a selected sample of 634 participants completed the WMH-CIDI-3.0 suicidality module (response rate 73%). ARMHS participants were selected for the WMH-CIDI-3.0 using stratified sampling according to psychological distress scores measured by Kessler-10 (K10) [20]: all participants with a score of 25 or greater (indicating high distress), three-quarters of those with a score from 16 to 24 (moderate distress) and 16.7% of participants scoring 10 to 15 (low psychological distress) were invited to participate in a telephone interview. ARMHS excluded residents of major cities. ARMHS and associated comparative analyses were approved by the Human Research Ethics Committees of the participating institutions: the University of Newcastle, University Sydney, Greater Western Area Health Service, Hunter New England Area Health Service and the North Coast Area Health Service.

### Measures

The WMH-CIDI-3.0 is a standardised diagnostic interview used to assess psychiatric diagnoses according to the Diagnostic and Statistical Manual (DSM-IV) [21] and International Classification of Diseases (ICD-10) [22] criteria. The WMH-CIDI-3.0 has been shown to have excellent inter-rater reliability, good test-retest reliability and validity and to be an acceptable method to determine lifetime psychiatric diagnoses [23]. The WMH-CIDI-3.0 includes a screening module and a number of diagnostic modules, including depressive, anxiety and substance use disorders, among others. The suicide module asks questions related to serious suicidal thoughts, suicide plans and suicide attempts, however participants screen out of the module if they answer “No” to the first question which asks ‘Have you ever seriously thought about committing suicide?’

### Geographical location variables

Parallel analyses of these two WMH-CIDI-3.0 datasets allowed comparison of the determinants of suicidal ideation and attempts across geographical regions by using the ABS Remoteness variable (RAO1CX). Household residence was categorised as: Major cities, Inner regional and Other [24]. The Other category combines Outer regional, Remote and Very remote regions from the Australian Geographical Classification System [25]. Data for the three individual components of this Other category were available for ARMHS, however not for the 2007-NSMHWB dataset; therefore, a more fine-grained analysis of remoteness was not possible. A separate ARMHS analysis was used to explore additional variables and sub-group differences, including comparisons between Inner regional, Outer Regional and Remote/very remote regions.

For the targeted age range (18–85 years), the 2007-NSMHWB data on remoteness made available by the ABS included 5,388 residents in Major cities, 1,943 in Inner regional areas and 1,132 in the rest of the state (Other). This population distribution is similar to that of the 2006 Australian Census data (see Table 1), with a small over-representation of regional residents. The ARMHS sample includes a greater proportion of residents from Outer regional, Remote and Very remote areas of New South Wales, as shown in Table 1.

**Table 1:** **Remoteness distribution across 2007-NSMHWB and ARMHS samples (aged 18 years and over, who completed the WMH-CIDI-3.0 suicidality module)**

Remoteness	Major cities	Inner regional	Other (Outer regional, remote & very remote)	Total
**Australian population (2006)** ^**¥**^	68.4% 14,159,826	19.7% 4,078,195	11.8% 2,442,777	100% 20,701,500
**2007-Australian NSMHWB**	63.7% 5,388	22.9% 1,943	13.4% 1,132	100% 8,463
**ARMHS WMH-CIDI-3.0 sample**	0.0% 0	39.6% 251	60.4% 383	100% 634

NSMHWB: National Survey of Mental Health and Well-being; ARMHS: Australian Rural Mental Health Study; WMH-CIDI-3.0: World Mental Health Composite International Diagnostic Interview version 3.0; Remoteness is measured using the Australian Bureau of Statistics (ABS) Remoteness variable (RAO1CX); ^¥^2006 Australian Census population data, accessed from http://www.abs.gov.au/AUSSTATS/abs@.nsf/Lookup/4102.0Chapter3002008.

### Characteristics

Demographic, socioeconomic, and health related variables of interest, common to both surveys, were identified as follows:

#### Demographic characteristics

Age was categorised as 18–44, 45–64 and 65–85 years. Gender was categorised as male or female and marital status was categorised as currently married versus not currently married. Level of education was categorised as university or higher degree versus no university degree, and employment was categorised as employed/not employed/or not in the workforce.

#### Socioeconomic characteristics

Financial adversity was measured by the number of financial adversity questions to which the respondent answered “Yes”, then categorised as low (0), moderate (1 or 2) or high (3 to 6) for analysis. The questions asked were: “Since January 2006 did any of the following happen to you because of a shortage of money?” 1) Could not pay electricity, gas or telephone bills on time; 2) Sold something; 3) Went without meals; 4) Unable to heat your home; 5) Sought financial help from friends or family; 6) Sought assistance from welfare/community organisations.

#### Physical health

Physical health was measured in terms of smoking status (currently smoking or not) and self-reported number of chronic diseases (heart disease, stroke, cancer or diabetes).

#### Mental health

Current psychological distress was measured by K10 [20], a 10-item measure of symptoms of distress during the previous four weeks, where scores range between 10 and 50 with higher scores indicating higher distress. The K10 has been shown to be sensitive to non-specific psychiatric distress [20, 26], and normative data for Australian populations have been developed [26, 27]. Mental disorder was assessed using the WMH-CIDI-3.0 and variables of interest included lifetime and 12-month diagnoses of any affective disorder, any anxiety disorder, any substance use disorder and any psychiatric disorder, or two or more lifetime psychiatric disorders (i.e., comorbidity). Service use for mental health problems was measured by self-reported contact with a mental health professional in the past 12 months (yes or no) and reporting that mental health services met their needs (yes or no).

### Outcome variables

The primary outcome variables were derived from the WHO-CIDI-3.0 Suicide module: experiencing serious suicidal thoughts in the past 12 months, reported as 12-month suicidal ideation; ever experiencing serious suicidal thoughts during your lifetime, reported as lifetime suicidal ideation; and ever attempting suicide, reported as lifetime suicide attempt. A sub-analysis was undertaken in the ARMHS sample for the outcome of lifetime suicidal ideation, incorporating the following additional variables that were not available as part of the 2007-NSMHWB:

#### Current functioning

A) The Well-being Index included seven scores in an aggregate measure: overall K10 score, days out of role in the past month, overall physical health, overall mental health, ability to perform everyday duties and tasks, satisfaction with relationships, and overall satisfaction with life [28], where higher scores indicate greater current well-being; B) the Alcohol Use Disorder Identification Test (AUDIT) [29], a 10 item measure of harmful or hazardous alcohol use during the previous six months, with a maximum total score of 40, where higher scores represent higher alcohol use and/or related harmful behaviours; C) Recent adverse life events were assessed using a 12-item measure of events in the past 12 months, including marital difficulties, becoming unemployed, experiencing a court case, or major financial difficulties [30].

#### Social support

The Community and Personal Support variable is a composite measure of five scales: perceived availability of social support [31], social networks [32], sense of community [33], community participation [34], and sense of place, which assesses connection with the local environment and landscape [35]. Higher scores indicate higher levels of support [36].

#### Personality

Personal optimism was measured by the 12-item version of the Hunter Opinions and Personal Expectations Scale (HOPES-12) [37, 38], administered to a sub-sample of baseline survey participants (n = 159). Neuroticism was assessed using the 12-item short form Eysenck Personality Inventory measure (EPI-12) [39], from which a 7-item subset was identified (i.e., being easily hurt, a nervous person, a worrier, being highly strung, suffering from nerves, worrying too long, and often guilty) that conceptually reflected pre-dispositional or trait characteristics, and may be usefully delineated from current distress items [28].

#### Socioeconomic factors

A) The Index of Relative Socio-Economic Advantage and Disadvantage (IRSAD) [40], a standardised score based on collation of household level census data that summarises information about the economic and social conditions of people and households within an area, including both relative advantage and disadvantage measures, where a score indicates a relative lack of disadvantage and greater advantage in general; B) Perceived prosperity was assessed using an item from the Household, Income and Labour Dynamics in Australia study asking about perceived prosperity (“Given your current needs and financial responsibilities, would you say that you and your family are: Prosperous, Very comfortable, Reasonably comfortable, Just getting along, or Poor?”) [41].

#### Physical health

Serious injury was assessed by asking participants to report whether or not they had experienced a serious injury in the last 12 months that required treatment from a doctor or hospital [42].

#### Service need and utilisation

A) Participants’ estimated need for professional mental health services was assessed using the Predicted Service Need Index [43]. This index is a composite of seven key measures of health and wellbeing: overall mental health; overall physical health; K10; AUDIT; Patient Health Questionnaire-9 [44]; recent adverse life events; and current smoking status. Scores on the index range from 0 to 14, with higher scores indicating a higher estimated need for mental health services; B) Actual use of mental health services in the previous 12-months was also reported.

#### Rural factors

A) Living on a farm – participants were asked to report if they currently lived on a farm; B) Proportion of time spent living in a rural area was assessed through the number of years residing in the current rural district; C) Infrastructure and services accessibility was assessed using four items specifically designed to reflect common concerns in rural communities (population change; access to health care or other services; concerns regarding fuel prices, people moving in or out of the district), with each item scored on a 5-point Likert scale ranging from ‘not at all’ to ‘a lot’; and D) Remoteness of residence was assessed using the Australian Standard Geographic Classification [25]: Major cities, Inner regional, Outer regional, Remote and Very remote. This sub-analysis combined Remote and Very remote categories.

### Statistical analysis

As the ‘Other locations’ categories were not directly comparable across the two datasets, due to differences in sampling strategies, and the non-availability of additional remoteness sub-categories for the ABS 2007-NSMHWB dataset, a parallel analysis strategy was adopted. That is, data from both samples were analysed independently, using SAS software version 9.2. Our 2007-NSMHWB dataset excluded participants aged less than 18 years. Proportions of participants reporting suicidal ideation and attempts were calculated for both samples across geographical regions. Demographic, socioeconomic, physical and mental health factors were initially compared between regions within samples, and across samples, for differences in proportions. Where appropriate, raw and back-weighted percentages are reported (e.g., the ARMHS interview data were back-weighted to represent the ARMHS baseline sample, to counteract the stratification of participants based on K10 psychological distress scores).

A series of logistic regression analyses were then undertaken for each of the primary outcomes of 12-month suicidal ideation, lifetime suicidal ideation and lifetime suicide attempts, reported as Adjusted Odds Ratios (AORs) and associated p-values. These analyses were not repeated for suicidal attempts in the last 12 months as relatively small proportions of those with suicidal ideation also reported a suicide attempt in this time period. Of note, interview questions about attempts were only initiated if ideation was reported. Due to the ARMHS sampling methods and survey response profiles [19, 28], all of the logistic analyses were adjusted for age, gender and psychological distress (K10) to minimise potential recruitment/selection biases. In practice, each predictor (or exposure) variable was included in a separate logistic regression, controlling for these three covariates; the decision to use a small, core set of covariates, versus inclusion in the regression models of all potential predictors, was based largely on the mixed nature of the predictor variables, which varied in their timeframes (e.g., lifetime factors, experiences during the past 12 months) and their levels of inherent interdependence (e.g., diagnostic status and health service consultations). Furthermore, the associations between the outcomes of interest and the potential predictor or exposure variables were investigated independently within each sample (2007-NSMHWB and ARMHS), including interactions with geographical location, expressed as Interaction Odds Ratios (IOR). A significant p-value for the IOR suggests that the effect of the predictor variable on the outcome of interest varies by location within that sample (i.e., across Major cities, Inner Regional and Other locations for the 2007-NSMHWB sample, or across Inner Regional versus Other locations for the ARMHS sample).

To reduce the likelihood of Type I errors (within each analysis series), we used Bonferroni-adjusted family-wise error rates, controlling for the number of predictor variables examined; for example, setting the threshold level of significance at p < 0.003 (i.e., .05/17) for the 17 individual predictors examined within the relevant regression analyses for a particular outcome and dataset.

## Results

A total of 634 participants completed the suicidality module of the WMH-CIDI-3.0 within ARMHS (251 from Inner regional and 383 from Other regions) and were compared to 5,388 urban residents and 3,075 non-urban residents from the 2007-NSMHWB sample (Table 2). This comparison indicated that the ARMHS sample included a higher proportion of female participants than the 2007-NSMHWB (61% vs. 55%, p = 0.002) and a lower proportion of younger participants (e.g., 22% vs. 48% in the 18–44 year age group, p < 0.001). Raw percentages from both samples plus back-weighted ARMHS data (with 99% Confidence intervals [CIs]) are shown in Table 2; the latter can be used to identify potential differences from the corresponding aggregate characteristics for the 2007-NSMHWB dataset. Additional file 1: Table S1 details actual participant numbers (i.e., cell sizes) for each of the characteristics examined – which are the denominators for the rates reported in subsequent tables.

**Table 2:** **Comparison of characteristics by region for 2007-NSMHWB and ARMHS samples**

Characteristic	Category	2007-NSMHWB raw %			ARMHS raw %		ARMHS back-weighted %	
		Major cities n = 5388	Inner regional n = 1943	Other n = 1132	Inner regional n = 251	Other n = 383	Inner regional n = 251 (99% CI)	Other n = 383 (99% CI)
Age in years	18-44	51	40	43	24	20	17 (9, 25)	21 (14, 28)
	45-64	28	34	32	54	54	50 (39, 62)	47 (38, 56)
	65-85	21	26	26	22	26	32 (21, 43)	32 (23, 40)
Gender	Female	55	54	55	59	62	57 (45, 68)	57 (48, 65)
Marital status	Married	46	51	47	63	64	69 (58, 79)	71 (63, 78)
Level of education	University degree	40	27	23	26	17	23 (13, 32)	18 (11, 24)
Employment status	Employed	64	59	59	50	55	49 (37, 61)	57 (48, 66)
	Unemployed	2.2	1.7	3.2	3.6	2.6	2.6 (0, 5.6)	1 (0.1, 1.8)
	Not in workforce	34	39	38	46	42	49 (37, 60)	42 (33, 51)
Financial adversity	Low	86	86	84	68	69	73 (63, 83)	79 (73, 86)
	Moderate	11	12	14	23	22	21 (11, 31)	15 (9, 21)
	High	2.2	2.7	2.3	8.6	8.5	5.5 (1.7, 9.4)	5.9 (2.2, 9.6)
Smoking (current status)	Yes	21	23	25	17	19	17 (8, 26)	14 (8, 20)
Chronic diseases (self-reported number)	0	69	60	63	56	56	56 (44, 68)	57 (48, 66)
	1	22	27	26	35	32	34 (23, 45)	32 (23, 40)
	2+	9.0	12	11	9.2	12	9.7 (2.8, 16.7)	12 (6, 17)
Any affective disorder	Lifetime	15	15	16	26	20	18 (11, 25)	13 (8, 18)
	12-month	6.0	5.8	6.0	16	9.9	7.7 (5.3, 10.1)	5.6 (3.1, 8)
Any anxiety disorder	Lifetime	26	26	27	45	39	39 (28, 50)	27 (20, 34)
	12 month	14	13	14	31	23	22 (13, 30)	13 (8, 17)
Any substance use disorder	Lifetime	23	25	30	23	26	20 (11, 29)	20 (14, 27)
	12-month	4.7	4.1	4.6	3.2	2.9	3.3 (0, 7.2)	1.4 (0.3, 2.5)
Any psychiatric disorder	Lifetime	51	55	55	65	64	56 (44, 67)	50 (41, 58)
Any psychiatric disorder	12-month	23	23	23	39	37	29 (19, 38)	21 (15, 26)
Two or more psychiatric disorders (comorbidity)	Yes	27	27	28	44	37	35 (25, 46)	25 (18, 31)
Any professional mental health service use^¥^	Yes	15	13	12	26	25	18 (11, 24)	19 (13, 24)
Did not get enough help^§^	Yes	19	14	18	38	37	31 (0, 65)	26 (3, 50)

NSMHWB: National Survey of Mental Health and Well-being; ARMHS: Australian Rural Mental Health Study, raw percentages and back-weighted for K10 stratification; see Additional file 1: Table S1 for cell sizes; CI: Confidence Intervals. ¥ Past 12 months; § Consulted a Mental Health professional in last 12 months and did not get enough help/info as needed. Assessments were based on WMH-CIDI-3.0: World Mental Health Composite International Diagnostic Interview version 3.0.

For the comparisons within samples, there were no statistically significant differences between Inner regional and Other participants for the ARMHS sample (using back-weighted data) for any characteristic. Within the 2007-NSMHWB sample, participant characteristics were similar for those living in Inner regional compared with Other regions, however those living in Major cities were younger (e.g., 51% vs. 41% in the 18–44 year age group, p < 0.001), had higher levels of education (e.g., 40% vs. 26% with a University or higher degree, p < 0.001) and employment (64% vs. 59% employed, p < 0.001), and lower chronic disease rates (31% vs. 39%, p < 0.001) when compared to Inner regional and Other regions.

As shown in Table 2 and Additional file 1: Table S1, for the comparison across samples for Inner regional areas, 2007-NSMHWB participants were younger than ARMHS (back-weighted) participants (e.g., 40% vs. 17% in the 18–44 year age group, p < 0.001). ARMHS participants were also more likely to be married (69% vs. 51%, p < 0.001), to have reported financial adversity (27% vs. 14%, p < 0.001), and to have met criteria for a lifetime anxiety disorder (39% vs. 26%, p < 0.001). For the comparison across samples for Other regions, 2007-NSMHWB participants were also more likely to be younger (e.g., 43% vs. 21% in the 18–44 year age group, p < 0.001), unemployed (3.2% vs. 1.0%, p = 0.010), smokers (25% vs. 14%, p < 0.001), and to have met criteria for lifetime substance use disorder (30% vs. 20%, p < 0.001). ARMHS participants in Other regions were more likely to be married (71% vs. 47%, p < 0.001), and to have used professional mental health services (19% vs. 12%, p < 0.001) (see Table 2 and Additional file 1: Table S1). No other differences were observed.

There were no statistically significant differences between regions (Inner regional compared to Other regions) or between samples (ARMHS compared to 2007-NSMHWB) for rates of lifetime or 12-month suicidal ideation or attempts, as shown in Table 3. Overall, 3.7% of the 2007-NSMHWB sample reported a lifetime suicide attempt compared with an estimated 5.8% of the ARMHS sample (p = 0.254). Further descriptors of suicidal behaviour across regions for the ARMHS (raw data) and the 2007-NSMHWB samples are shown in Additional file 2: Table S2.

**Table 3:** **Percentage of participants reporting lifetime and 12 month suicidal ideation and attempts in 2007-NSMHWB and ARMHS samples**

Variable	2007-NSMHWB raw %			ARMHS raw %		ARMHS back-weighted % (99% CI)		Chi square p-value
	Major cities n = 5388	Inner regional n = 1943	Other n = 1132	Inner regional n = 251	Other n = 383	Inner regional n = 251	Other n = 383	
Lifetime suicidal ideation	14	15	16	26	23	19 (11.2, 27.2)	15 (9.5, 19.6)	0.254
12-month suicidal ideation	2.7	2.5	2.6	8.8	4.2	5.1 (1.7, 8.5)	1.9 (0.7, 3.2)	0.864
Lifetime suicide attempt	3.6	3.7	4.7	8.0	7.0	7.2 (1.6, 12.8)	4.8 (1.7, 7.9)	0.202
12-month suicide attempt	0.4	0.3	0.8	1.2	0.8	0.7 (0, 1.7)	0.4 (0, 1.0)	0.093

NSMHWB: National Survey of Mental Health and Well-being; ARMHS: Australian Rural Mental Health Study, raw percentages and back-weighted for K10 stratification (i.e., to adjust for selection strategy); CI: Confidence Intervals. Assessments were based on WMH-CIDI-3.0: World Mental Health Composite International Diagnostic Interview version 3.0.

### 12-month suicidal ideation

Logistic regression analyses for the outcome of 12-month suicidal ideation (adjusted for age, gender and K10 psychological distress) in both samples are shown in Table 4. Factors associated with 12-month suicidal ideation across the two samples, analysed independently, were broadly similar, with similar effect sizes. The common factors across samples that significantly increased the odds of experiencing 12-month suicidal ideation were mental health factors including high psychological distress (AORs of 50 and 36, p < 0.001), any 12-month affective, anxiety or substance use disorder (AORs ranging from 2.0 to 11, p < 0.001), and any 12-month psychiatric disorder or multiple disorders (AORs ranging from 1.9 to 6.8, p < 0.001). In the NSMHWB sample only, the adjusted odds of 12-month suicidal ideation were lower in married persons (AOR 0.43, p < 0.001), and higher in those with moderate psychological distress (AOR 9.7, p < 0.001), moderate or high financial adversity (AORs of 1.7 and 2.9, p < 0.001), any lifetime psychiatric disorder (AOR 2.0, p = 0.001), and any mental health professional service use in the past 12 months (AOR 4.1, p < 0.001).

**Table 4:** **Selected logistic regressions for 12-month suicidal ideation by remoteness, comparing 2007-NSMHWB and ARMHS samples**

Characteristic (predictor or exposure variable)	Category	2007-NSMHWB ^#^					ARMHS ^#^			
		Major cities (n = 5388) n (%)	Inner Regional (n = 1943) n (%)	Other (n = 1132) n (%)	AOR	p-value	Inner Regional (n = 251) n (%)	Other (n = 383) n (%)	AOR	p-value
**Demographic factors** Age in years										
	18-44	93 (3.4)	20 (2.5)	15 (3.1)	.	.	9 (15)	5 (6.4)	.	.
	45-64	41 (2.7)	19 (2.9)	10 (2.8)	0.87	0.381	11 (8.1)	8 (3.9)	0.57	0.143
	65-85	11 (1.0)	9 (1.8)	4 (1.4)	0.58	0.023	2 (3.6)	3 (3.0)	0.40	0.105
Gender	Male	53 (2.2)	24 (2.7)	14 (2.7)	.	.	10 (9.7)	6 (4.1)	.	.
	Female	92 (3.1)	24 (2.3)	15 (2.4)	0.93	0.606	12 (8.1)	10 (4.2)	0.83	0.596
Marital status	Not married	116 (4.0)	34 (3.6)	25 (4.1)	.	.	12 (13)	9 (6.6)	.	.
	Currently married	29 (1.2)	14 (1.4)	4 (0.8)	**0.43**	**<0.001**	10 (6.3)	7 (2.9)	0.65	0.218
Education	No university degree	92 (2.8)	38 (2.7)	18 (2.1)	.	.	17 (9.2)	12 (3.8)	.	.
	University degree	53 (2.5)	10 (1.9)	11 (4.2)	1.0	0.969	5 (7.6)	4 (6.2)	1.1	0.805
Employment status	Employed	78 (2.3)	21 (1.8)	15 (2.2)	.	.	9 (7.2)	9 (4.3)	.	.
	Not in Workforce	7 (5.9)	1 (3.0)	3 (8.3)	1.5	0.011	10 (8.6)	7 (4.3)	1.6	0.249
	Unemployed	60 (3.3)	26 (3.4)	11 (2.6)	1.8	0.095	3 (33)		2.0	0.338
Financial adversity	Low	88 (1.9)	26 (1.6)	12 (1.3)	.	.	7 (4.9)	7 (3.2)		.
	Moderate	38 (6.2)	14 (6.3)	12 (7.7)	**1.7**	**0.001**	7 (15)	4 (5.7)	1.7	0.217
	High	19 (16)	8 (15)	5 (19)	**2.9**	**<0.001**	3 (17)	3 (11)	1.3	0.601
**Physical health**										
Smoking	No	85 (2.0)	31 (2.1)	18 (2.1)	.	.	15 (8.5)	8 (3.1)		.
	Yes	60 (5.4)	17 (3.8)	11 (3.9)	1.4	0.033	3 (8.1)	7 (12)	1.1	0.794
Number of chronic diseases	0	101 (2.7)	26 (2.2)	22 (3.1)	.	.	13 (9.2)	10 (4.6)	.	.
	1	27 (2.2)	14 (2.6)	3 (1.0)	0.85	0.391	6 (6.9)	5 (4.1)	1.0	0.988
	> = 2	17 (3.5)	8 (3.4)	4 (3.1)	1.4	0.161	3 (13)	1 (2.2)	1.2	0.787
**Mental health**										
Psychological distress (K10)	Low	20 (0.5)	3 (0.2)	5 (0.6)	.	.	1 (1.4)		.	.
	Moderate	59 (4.8)	18 (4.1)	13 (5.2)	**9.7**	**<0.001**	7 (5.4)	8 (4.2)	8.9	0.036
	High	66 (20)	26 (22)	10 (18)	**50**	**<0.001**	14 (27)	8 (11)	**36**	**<0.001**
Any affective disorder	No lifetime diagnosis	70 (1.5)	22 (1.3)	11 (1.2)	.	.	7 (3.8)	6 (2.0)		.
	Lifetime diagnosis with 12-month symptoms	63 (20)	21 (19)	14 (21)	**3.9**	**<0.001**	13 (33)	6 (16)	**5.5**	**<0.001**
	Lifetime diagnosis with no 12-month symptoms	12 (2.5)	5 (2.9)	4 (3.7)	0.97	0.895	2 (7.7)	4 (10)	2.7	0.057
Any anxiety disorder	No lifetime diagnosis	58 (1.5)	20 (1.4)	11 (1.3)	.	.	5 (3.6)	3 (1.3)	.	.
	Lifetime diagnosis with 12-month symptoms	75 (10)	23 (9.0)	18 (11)	**2.0**	**<0.001**	16 (21)	8 (9.1)	**4.3**	**0.001**
	Lifetime diagnosis with no 12 month symptoms	12 (1.8)	5 (2.0)	0 (0.0)	0.62	0.083	1 (2.7)	5 (7.9)	2.4	0.121
Any substance use disorder	No lifetime diagnosis	88 (2.1)	24 (1.7)	15 (1.9)	.	.	13 (6.7)	7 (2.5)	.	.
	Lifetime diagnosis with 12 month symptoms	29 (11)	9 (11)	7 (13)	**2.9**	**<0.001**	4 (50)	5 (45)	**11**	**<0.001**
	Lifetime diagnosis with no 12 month symptoms	28 (2.9)	15 (3.7)	7 (2.5)	1.1	0.623	5 (10)	4 (4.6)	1.1	0.898
Any lifetime psychiatric disorder	No	24 (0.9)	4 (0.5)	3 (0.6)	.	.		1 (0.7)	.	.
	Yes	121 (4.4)	44 (4.1)	26 (4.2)	**2.0**	**0.001**	22 (13)	15 (6.1)	11	0.020
Any 12 month psychiatric disorder	No	38 (0.9)	11 (0.7)	7 (0.8)	.	.	4 (2.6)	1 (0.4)		.
	Yes	107 (8.7)	37 (8.2)	22 (8.5)	**3.5**	**<0.001**	18 (18)	15 (11)	**6.8**	**<0.001**
Two or more psychiatric disorders	No	50 (1.3)	14 (1.0)	9 (1.1)	.	.	3 (2.1)	4 (1.6)	.	.
	Yes	95 (6.6)	34 (6.4)	20 (6.3)	**1.9**	**<0.001**	19 (18)	12 (8.6)	**4.3**	**0.001**
**Health service use past 12 months**										
Any professional mental health service use	No	57 (1.2)	15 (0.9)	12 (1.2)	.	.	10 (5.5)	7 (2.5)	.	.
	Yes	88 (11)	33 (13)	17 (12)	**4.1**	**<0.001**	12 (17)	9 (8.7)	2.0	0.048
Consulted a mental health professional and did not get enough help/info as needed	No, needs met	41 (12)	16 (15)	8 (15)	.	.	6 (13)	4 (5.6)	.	.
	Yes, unmet need	24 (29)	6 (35)	2 (17)	1.8	0.023	6 (23)	6 (16)	2.0	0.162

NSMHWB: National Survey Mental Health and Well-being (aged 18–85); ARMHS: Australian Rural Mental Health Study.

^#^Bracketed values refer to the percentage of each predictor variable sub-category reporting 12-month suicidal ideation; see Additional file 1: Table S1 for cell sizes.

Note: Each predictor variable was included in a separate logistic regression, controlling for age, gender, and K10 psychological distress score (as appropriate); AOR: Adjusted Odds Ratio - adjusted for the covariates; bolded p-values are statistically significant (against Bonferroni-adjusted thresholds); see Additional file 3: Tables S3a and Additional file 3: Table S3b for AOR Confidence Intervals and (non-significant) Predictor variable x Region interactions.

### Lifetime suicidal ideation

For the outcome of lifetime suicidal ideation (adjusted for age, gender and K10 psychological distress) the results for both samples are shown in Table 5. The factors associated with lifetime suicidal ideation were also similar across samples. Among the demographic variables there were statistically significantly lower odds of lifetime suicidal ideation for those currently married (AORs ranging from 0.48 to 0.54, p < 0.001). Among the mental health factors, those reporting moderate to high psychological distress (AORs ranging from 2.7 to 11, p < 0.001), those with any lifetime or 12-month affective disorder (AORs ranging from 2.6 to 7.1, p < 0.001), or substance use disorders (AORs ranging from 2.0 to 6.7, p < 0.001), those with 12-month anxiety disorders (AORs ranging from 2.7 to 3.6, p < 0.001), any lifetime or 12-month psychiatric disorder (AORs ranging from 2.3 to 4.7, p < 0.001), two or more psychiatric disorders (AOR 3.2 to 4.6, p < 0.001) and those using mental health services (AORs of 2.1 to 3.6, p < 0.001), increased the odds of reporting lifetime suicidal ideation for both samples.

**Table 5:** **Selected logistic regressions for lifetime suicidal ideation by remoteness, comparing 2007-NSMHWB and ARMHS samples**

Characteristic (predictor or exposure variable)	Category	2007-NSMHWB ^#^					ARMHS ^#^			
		Major cities (n = 5388) n (%)	Inner regional (n = 1943) n (%)	Other (n = 1132) n (%)	AOR	p-value	Inner regional (n = 251) n (%)	Other (n = 383) n (%)	AOR	p-value
**Demographic factors**										
Age in years	18-44	401 (15)	121 (15)	91 (19)	.	.	25 (42)	22 (28)	.	.
	45-64	268 (18)	128 (19)	66 (18)	**1.3**	**<0.001**	36 (27)	48 (23)	0.68	0.095
	65-85	87 (7.8)	40 (8.0)	22 (7.5)	**0.59**	**<0.001**	5 (8.9)	18 (18)	0.43	0.005
Gender	Male	310 (13)	114 (13)	79 (15)	.	.	27 (26)	26 (18)	.	.
	Female	446 (15)	175 (17)	100 (16)	1.1	0.254	39 (26)	62 (26)	1.2	0.283
Marital status	Not married	529 (18)	185 (20)	120 (20)	.	.	31 (33)	51 (37)	.	.
	Currently married	227 (9.2)	104 (10)	59 (11)	**0.54**	**<0.001**	35 (22)	36 (15)	**0.48**	**<0.001**
Education	No university degree	471 (15)	221 (16)	130 (15)	.	.	49 (26)	72 (23)	.	.
	University or higher degree	285 (13)	68 (13)	49 (19)	0.87	0.056	17 (26)	16 (25)	0.94	0.784
Employment status	Employed	480 (14)	164 (14)	97 (14)	.	.	30 (24)	44 (21)	.	.
	Not in Workforce	32 (27)	6 (18)	8 (22)	1.3	0.004	30 (26)	42 (26)	1.9	0.006
	Unemployed	244 (13)	119 (16)	74 (17)	1.5	0.021	5 (56)	2 (20)	1.4	0.492
Financial adversity	Low	536 (12)	210 (13)	121 (13)	.	.	25 (17)	41 (19)	.	.
	Moderate	157 (26)	56 (25)	48 (31)	**1.7**	**<0.001**	22 (46)	20 (29)	1.9	0.009
	High	63 (54)	23 (44)	10 (38)	**3.5**	**<0.001**	6 (33)	13 (48)	1.6	0.223
**Physical health**										
Smoking	No	507 (12)	181 (12)	104 (12)	.	.	43 (24)	53 (20)	.	.
	Yes	249 (22)	108 (24)	75 (26)	**1.8**	**<0.001**	12 (32)	22 (37)	1.2	0.444
Number of chronic diseases	0	492 (13)	159 (14)	109 (15)	.	.	43 (30)	60 (28)		.
	1	177 (15)	82 (15)	46 (16)	1.3	0.004	18 (21)	21 (17)	0.63	0.055
	> = 2	87 (18)	48 (21)	24 (19)	**2.0**	**<0.001**	5 (22)	7 (15)	0.58	0.164
**Mental health**										
Psychological distress (K10)	Low	308 (8.1)	115 (8.3)	85 (10)	.	.	9 (13)	9 (7.6)	.	.
	Moderate	282 (23)	110 (25)	64 (26)	**3.3**	**<0.001**	30 (23)	46 (24)	**2.7**	**<0.001**
	High	166 (50)	63 (53)	30 (53)	**11**	**<0.001**	27 (52)	33 (45)	**7.9**	**<0.001**
Any affective disorder	No lifetime diagnosis	397 (8.7)	158 (9.5)	95 (9.9)	.	.	29 (16)	47 (15)	.	.
	Lifetime diagnosis with 12 month symptoms	157 (49)	59 (53)	31 (46)	**4.2**	**<0.001**	23 (59)	16 (42)	**2.6**	**0.001**
	Lifetime diagnosis with no 12 month symptoms	202 (42)	72 (42)	53 (49)	**5.8**	**<0.001**	14 (54)	25 (63)	**7.1**	**<0.001**
Any anxiety disorder	No lifetime diagnosis	308 (7.7)	125 (8.7)	76 (9.2)	.	.	19 (14)	36 (16)	.	.
	Lifetime diagnosis with 12 month symptoms	278 (37)	100 (39)	61 (38)	**3.6**	**<0.001**	37 (48)	33 (38)	**2.7**	**<0.001**
	Lifetime diagnosis with no 12 month symptoms	170 (26)	64 (25)	42 (28)	**3.2**	**<0.001**	10 (27)	19 (30)	2.1	0.009
Any substance use disorders	No lifetime diagnosis	438 (11)	170 (12)	96 (12)	.	.	39 (20)	53 (19)	.	.
	Lifetime diagnosis with 12 month symptoms	86 (34)	23 (29)	13 (25)	**2.6**	**<0.001**	6 (75)	7 (64)	**6.7**	**<0.001**
	Lifetime diagnosis with no 12 month symptoms	232 (24)	96 (23)	70 (25)	**2.5**	**<0.001**	21 (42)	28 (32)	**2.0**	**0.003**
Any lifetime psychiatric disorder	No	107 (4.0)	38 (4.3)	28 (5.5)	.	.	4 (4.5)	11 (8.1)	.	.
	Yes	649 (24)	251 (24)	151 (24)	**4.7**	**<0.001**	62 (38)	77 (31)	**4.7**	**<0.001**
Any 12 month psychiatric disorder	No	344 (8.3)	143 (9.6)	98 (11)	.	**.**	22 (14)	36 (15)	.	.
	Yes	412 (33)	146 (33)	81 (31)	**2.8**	**<0.001**	44 (44)	52 (37)	**2.3**	**<0.001**
Two or more psychiatric disorders	No	262 (6.6)	109 (7.7%)	63 (7.7)	.	.	17 (12)	34 (14)	.	.
	Yes	494 (35)	180 (34)	116 (37)	**4.6**	**<0.001**	49 (45)	54 (39)	**3.2**	**<0.001**
**Health service use in past 12 months**										
Any professional mental health service use	No	445 (9.7)	173 (10)	115 (12)	.	.	37 (20)	47 (17)	.	.
	Yes	311 (39)	116 (44)	64 (46)	**3.6**	**<0.001**	29 (41)	41 (39)	**2.1**	**<0.001**
Consulted a mental health professional and did not get as much help/info as needed	No, needs met	120 (34)	46 (43)	21 (40)	.	.	17 (35)	27 (38)	.	.
	Yes, unmet need	50 (60)	10 (59)	6 (50)	1.7	0.019	14 (54)	18 (49)	1.4	0.291

NSMHWB: National Survey Mental Health and Well-being (aged 18–85); ARMHS: Australian Rural Mental Health Study.

^#^Bracketed values refer to the percentage of each predictor (or exposure) variable sub-category reporting lifetime suicidal ideation; see Additional file 1: Table S1 for cell sizes.

Note: Each predictor variable was included in a separate logistic regression, controlling for age, gender, and K10 psychological distress score (as appropriate); AOR: Adjusted Odds Ratio - adjusted for the covariates; bolded p-values are statistically significant (against Bonferroni-adjusted thresholds); see Additional file 4: Table S4a and Additional file 4: Table S4b for AOR Confidence Intervals and (non-significant) Predictor variable x Region interactions.

In the NSMHWB sample only, compared with those aged 18–44 years, there were higher odds of lifetime suicidal ideation in those aged 45–64 years (AOR 1.3, p < 0.001) and lower odds in those aged 65–85 years (AOR 0.59, p < 0.001). In terms of the physical health factors, current smoking (AOR 1.8, p < 0.001) and reporting two or more chronic diseases (AOR 2.0, p <0.001) were associated with lifetime suicidal ideation as was reporting moderate to high financial adversity (AORs 1.7 and 3.5, p < 0.001). For mental health factors, any lifetime anxiety disorder without 12 months symptoms was associated with increased odds of lifetime suicidal ideation (AOR 3.2, p < 0.001).

### Lifetime suicide attempts

For the outcome of lifetime suicide attempts (adjusted for age, gender and K10 psychological distress) the results for both samples are shown in Table 6. The only demographic factor statistically significantly associated with lower odds of lifetime suicide attempts across both samples was being currently married (AORs ranging from 0.29 to 0.36, p < 0.001). The mental health factors statistically significantly associated with higher odds of lifetime suicide attempts across both samples were current high psychological distress (AOR ranging from 4.7 to 12, p < 0.001) and those with any lifetime or 12-month affective disorder (AORs ranging from 4.0 to 6.7, p < 0.001), any anxiety disorder with 12-month symptoms (AOR of 6.0, p < 0.001), any substance use disorder (AORs ranging from 3.1 to 5.2, p < 0.003), any 12-month psychiatric disorder (AORs ranging from 3.5 to 5, p < 0.001), and having two or more lifetime psychiatric disorders (AORs ranging from 5.3 to 8.1, p < 0.001).

**Table 6:** **Selected logistic regressions for lifetime suicide attempts by remoteness comparing 2007-NSMHWB and ARMHS samples**

Characteristic (predictor or exposure variable)	Category	2007-NSMHWB ^#^					ARMHS ^#^			
		Major cities (n = 5388) n (%)	Inner regional (n = 1943) n (%)	Other (n = 1132) n (%)	AOR	p-value	Inner regional (n = 251) n (%)	Other (n = 383) n (%)	AOR	p-value
**Demographic factors**										
Age in years	18-44	118 (4.3)	33 (4.2)	25 (5.2)	.	.	9 (15)	9 (12)	.	.
	45-64	63 (4.1)	28 (4.2)	23 (6.4)	1.1	0.560	10 (7.4)	15 (7.3)	0.58	0.103
	65-85	12 (1.1)	10 (2.0)	5 (1.7)	**0.42**	**<0.001**	1 (1.8)	3 (3.0)	0.21	0.007
Gender	Male	65 (2.7)	24 (2.7)	17 (3.3)	.	.	10 (9.7)	7 (4.8)	.	.
	Female	128 (4.3)	47 (4.5)	36 (5.8)	**1.4**	**0.003**	10 (6.8)	20 (8.5)	1.1	0.759
Marital status	Not married	154 (5.3)	56 (5.9)	40 (6.6)	.	.	13 (14)	19 (14)	.	.
	Currently married	39 (1.6)	15 (1.5)	13 (2.5)	**0.36**	**<0.001**	7 (4.4)	8 (3.3)	**0.29**	**<0.001**
Education	No university degree	129 (4.0)	54 (3.8)	44 (5.0)	.	.	15 (8.1)	21 (6.6)	.	.
	University or higher degree	64 (3.0)	17 (3.2)	9 (3.5)	0.69	0.004	5 (7.6)	6 (9.2)	1.0	0.956
Employment status	Employed	119 (3.4)	31 (2.7)	26 (3.9)	.	.	5 (4.0)	13 (6.3)	.	.
	Not in Workforce	61 (3.4)	37 (4.9)	25 (5.9)	1.5	0.004	11 (9.5)	13 (8.1)	**3.0**	**0.002**
	Unemployed	13 (11)	3 (9.1)	2 (5.6)	**2.3**	**0.002**	4 (44)	1 (10)	5.0	0.009
Financial adversity	Low	116 (2.5)	44 (2.6)	30 (3.2)	.	.	5 (3.5)	10 (4.5)	.	.
	Moderate	49 (8.0)	16 (7.1)	18 (12)	**1.9**	**<0.001**	8 (17)	8 (11)	3.0	0.005
	High	28 (24)	11 (21)	5 (19)	**4.3**	**<0.001**	4 (22)	5 (19)	2.9	0.036
**Physical health**										
Smoking	No	104 (2.4)	37 (2.5)	22 (2.6)	.	.	11 (6.3)	13 (5.0)	.	.
	Yes	89 (8.0)	34 (7.7)	31 (11)	**2.7**	**<0.001**	7 (19)	9 (15)	2.1	0.051
Number of chronic diseases	0	127 (3.4)	46 (3.9)	34 (4.8)	.	.	14 (9.9)	19 (8.8)	.	.
	1	48 (4.0)	17 (3.2)	12 (4.1)	1.3	0.294	4 (4.6)	5 (4.1)	0.60	0.218
	> = 2	18 (3.7)	8 (3.4)	7 (5.5)	1.3	0.160	2 (8.7)	3 (6.5)	1.5	0.502
**Mental health**										
Psychological distress (K10)	Low	62 (1.6)	23 (1.7)	16 (1.9)	.	.	4 (5.7)	3 (2.5)	.	.
	Moderate	71 (5.8)	28 (6.3)	24 (9.6)	**3.7**	**<0.001**	6 (4.7)	12 (6.3)	1.3	0.527
	High	60 (18)	20 (17)	13 (23)	**12**	**<0.001**	10 (19)	12 (16)	**4.7**	**<0.001**
Any affective disorder	No lifetime diagnosis	72 (1.6)	27 (1.6)	23 (2.4)	.	.	6 (3.2)	11 (3.6)	.	.
	Lifetime diagnosis with 12 month symptoms	56 (17)	20 (18)	14 (21)	**5.1**	**<0.001**	11 (28)	10 (26)	**6.7**	**<0.001**
	Lifetime diagnosis with no 12 month symptoms	65 (13)	24 (14)	16 (15)	**6.4**	**<0.001**	3 (12)	6 (15)	**4.0**	**0.002**
Any anxiety disorder	No lifetime diagnosis	53 (1.3)	18 (1.3)	13 (1.6)	.	.	2 (1.5)	7 (3.0)	**.**	.
	Lifetime diagnosis with 12 month symptoms	96 (13)	39 (15)	30 (19)	**6.0**	**<0.001**	15 (19)	14 (16)	**6.0**	**<0.001**
	Lifetime diagnosis with no 12 month symptoms	44 (6.7)	14 (5.5)	10 (6.8)	**3.8**	**<0.001**	3 (8.1)	6 (9.5)	3.6	0.010
Any substance use disorders	No lifetime diagnosis	81 (1.9)	35 (2.4)	23 (2.9)	.	.	8 (4.1)	13 (4.6)	.	.
	Lifetime diagnosis with 12 month symptoms	35 (14)	11 (14)	7 (13)	**5.2**	**<0.001**	3 (38)	3 (27)	**6.1**	**0.003**
	Lifetime diagnosis with no 12 month symptoms	77 (7.9)	25 (6.1)	23 (8.1)	**3.7**	**<0.001**	9 (18)	11 (13)	**3.1**	**0.001**
Any lifetime psychiatric disorder	No	12 (0.5)	5 (0.6)	5 (1.0)	.	.	1 (1.1)		.	.
	Yes	181 (6.6)	66 (6.2)	48 (7.7)	**7.7**	**<0.001**	19 (12)	27 (11)	19	0.004
Any 12 month psychiatric disorder	No	67 (1.6)	26 (1.7)	17 (1.9)	.	.	3 (2.0)	7 (2.9)	.	.
	Yes	126 (10)	45 (10)	36 (14)	**3.5**	**<0.001**	17 (17)	20 (14)	**5.0**	**<0.001**
Two or more psychiatric disorders	No	39 (1.0)	12 (0.8)	8 (1.0)	.	.	2 (1.4)	7 (2.9)	.	.
	Yes	154 (11)	59 (11)	45 (14)	**8.1**	**<0.001**	18 (17)	20 (14)	**5.3**	**<0.001**
**Health service use in past 12 months**										
Any professional mental health service use	No	77 (1.7)	23 (1.4)	21 (2.1)	.	.	8 (4.4)	14 (5.0)	.	.
	Yes	116 (14)	48 (18)	32 (23)	**6.5**	**<0.001**	12 (17)	13 (13)	2.5	0.007
Consulted a mental health professional and did not get as much help/info as needed	No, needs met	46 (13)	21 (20)	12 (23)	.	.	6 (13)	7 (9.7)	.	.
	Yes, unmet need	23 (27)	4 (24)	3 (25)	1.4	0.230	8 (31)	8 (22)	2.1	0.086

NSMHWB: National Survey Mental Health and Well-being (aged 18–85); ARMHS: Australian Rural Mental Health Study.

^#^Bracketed values refer to the percentage of each predictor (or exposure) variable sub-category reporting lifetime suicide attempt; see Additional file 1: Table S1 for cell sizes.

Note: Each predictor variable was included in a separate logistic regression, controlling for age, gender, and K10 psychological distress score (as appropriate); AOR: Adjusted Odds Ratio - adjusted for the covariates; bolded p-values are statistically significant (against Bonferroni-adjusted thresholds); see Additional file 5: Table S5a and Additional file 5: Table S5b for AOR Confidence Intervals and (non-significant) Predictor variable x Region interactions.

In the NSMHWB sample only, older age reduced the odds of a lifetime suicide attempt (AOR 0.42, p < 0.001) and being unemployed (AOR 2.3, p = 0.002) or experiencing moderate or high financial adversity (AORs 1.9 to 4.3, p < 0.001) increased the odds of a lifetime suicide attempt. In terms of mental health factors in the NSMHWB, experiencing current moderate psychological distress, having any lifetime anxiety disorder without 12-month symptoms, and any lifetime psychiatric disorder increased the odds of a lifetime suicide attempt (AORs of 3.7, 3.8 and 7.7, p < 0.001 respectively). Reporting any professional mental health service use in the past 12 months also statistically significantly increased the odds of a lifetime suicide attempt (AOR 6.5, p < 0.007) in the NSMHWB sample.

### Suicidal ideation across geographical regions

There were no statistically significant interaction effects for geographical location with any predictor variable tested for the outcomes of 12-month or lifetime suicidal ideation or lifetime suicide attempt (IORs and 99% CIs are shown in Additional file 3: Table S3a, Additional file 4: Table S4a and Additional file 5: Table S5a for the 2007-NSMHWB sample, and S3b, S4b and S5b for the ARMHS sample); that is, the observed associations between the predictor variables and the outcomes of interest reported above did not vary by geographical location (within samples).

### Additional comparisons and regression analyses within the ARMHS sample

As there were additional variables collected in the ARMHS sub-sample, which were not available in the national dataset, we undertook some further analyses. Firstly, we classified the 634 ARMHS participants into three discrete groups: no lifetime suicidal ideation (76%); lifetime ideation, but not recently (18%); and ideation during the last 12-months (5.9%) in order to assess the influence of the additional ARMHS variables reporting current functioning and social support. The left-hand columns of Table 7 present simple (univariate) profiles for these groups; overall statistical tests are reported (chi-square tests for the categorical variables and one-way ANOVAs for the continuous measures). In short, those with lifetime suicidal ideation tended to be: younger, not married, with higher psychological distress, lower wellbeing, more recent adverse events, lower community and personal support, lower optimism, higher neuroticism, greater financial adversity, greater perceived need for services, and greater service use for mental health. The reporting of a lifetime psychiatric disorder (any affective disorder, any anxiety disorder and any substance use disorder) was also significantly associated with lifetime suicidal ideation at the univariate level.

**Table 7:** **Additional comparisons and expanded logistic regressions for lifetime suicidal ideation in ARMHS sample (n = 634)**

Characteristic (predictor or exposure variable)	Statistic or category	Profiles for discrete ARMHS sub-groups				Prediction of lifetime suicidal ideation ^#^	
		No lifetime suicidal ideation (n = 480)	Lifetime suicidal ideation		Overall univariate analysis p-value	AOR (99% CI)	p-value
			Not recent (n = 116)	Present during last 12-months (n = 38)			
**Demographics**							
Age in years	mean (SD, n)	57 (14, 480)	52 (13, 116)	48 (15, 38)	**<0.001**	**0.98** (0.96, 1.0)	**0.001**
Gender	Female	283 (59%)	79 (68%)	22 (58%)	0.183	1.18 (0.68, 2.0)	0.445
Marital status	Yes	330 (69%)	54 (47%)	17 (45%)	**<0.001**	**0.51** (0.30, 0.87)	**0.001**
Education	University or higher degree	98 (20%)	24 (21%)	9 (24%)	0.892	0.90 (0.47, 1.7)	0.671
**Current functioning**							
Current psychological distress (K10)	mean (SD, n)	18 (6.3, 480)	21 (6.4, 116)	27 (7.8, 38)	**<0.001**	**1.07** (1.03, 1.1)	**<0.001**
Wellbeing index	mean (SD, n)	-.32 (0.82, 480)	-.70 (0.93, 115)	−1.3 (0.85, 38)	**<0.001**	0.88 (0.54, 1.4)	0.485
Alcohol Use (AUDIT)	mean (SD, n)	3.7 (3.9, 480)	3.8 (4.9, 116)	6.3 (6.9, 38)	0.004	1.0 (0.97, 1.1)	0.226
Adverse life events	mean (SD, n)	1.7 (1.6, 480)	2.2 (1.9, 116)	3.1 (2.1, 38)	**<0.001**	1.1 (0.94, 1.3)	0.138
**Social support**							
Community & personal support	mean (SD, n)	−0.2 (0.8, 467)	−0.4 (0.8, 115)	−1.0 (0.9, 37)	**<0.001**	0.88 (0.60, 1.3)	0.355
**Personality**							
Optimism (HOPES-12)	mean (SD, n)	2.8 (0.6, 121)	2.3 (0.8, 28)	2.1 (0.7, 10)	**<0.001**	**0.21** (0.06, 0.68)	**<0.001**
Neuroticism	mean (SD, n)	2.6 (2.0, 468)	3.3 (2.2, 115)	4.2 (2.3, 36)	**<0.001**	1.0 (0.89, 1.2)	0.563
**Socioeconomic factors**							
SEIFA (IRSAD)	mean (SD)	939.03 (38.70)	943.22 (39.81)	946.76 (36.21)	0.329	1.0 (1.00, 1.0)	0.168
Financial adversity	Low	298 (75%)	52 (54%)	14 (45%)	**<0.001**		
	Moderate	76 (19%)	31 (32%)	11 (35%)		1.8 (0.91, 3.4)	0.028
	High	26 (6.5%)	13 (14%)	6 (19%)		1.3 (0.50, 3.6)	0.432
**Physical illness**							
Smoker	Yes	63 (16%)	24 (25%)	10 (30%)	0.021	1.1 (0.54, 2.2)	0.748
No. of chronic illnesses	0	254 (53%)	80 (69%)	23 (61%)	0.037		
	1	169 (35%)	11 (29%)	28 (24%)		0.67 (0.36, 1.3)	0.105
	2+	57 (12%)	4 (11%)	8 (6.9%)		0.59 (0.22, 1.6)	0.176
Serious injury past 12 months	Yes	61 (13%)	7 (18%)	25 (22%)	0.043	1.5 (0.74, 3.0)	0.148
**Service need and utilization**							
Perceived need for services	mean (SD, n)	3.0 (3.0, 480)	4.9 (3.5, 116)	7.7 (3.2, 38)	**<0.001**	1.14 (1.01, 1.3)	0.006
Service use for mental health problem	Yes	93 (19%)	46 (40%)	21 (55%)	**<0.001**	**2.12** (1.21, 3.7)	**<0.001**
**Rural factors**							
Live on farm	Yes	116 (25%)	23 (20%)	8 (21%)	0.576	0.89 (0.47, 1.7)	0.639
Proportion of life in a rural area	mean (SD, n)	0.70 (0.31, 475)	0.67 (0.30, 113)	0.73 (0.31, 38)	0.573	0.84 (0.36, 1.9)	0.601
Remoteness category	Inner regional	185 (39%)	44 (38%)	22 (58%)	0.029	.	
	Outer regional	175 (36%)	53 (46%)	10 (26%)		1.09 (0.61, 1.9)	0.698
	Remote/ Very Remote	120 (25%)	19 (16%)	6 (16%)		0.63 (0.30, 1.3)	0.11
Infrastructure and services accessibility	mean (SD, n)	4.00 (3.15, 480)	3.30 (2.55, 116)	3.03 (2.31, 38)	0.021	0.91 (0.82, 1.0)	0.007
**Lifetime psychiatric morbidity**							
Affective disorder	Yes	65 (14%)	53 (46%)	30 (79%)	**<0.001**	**3.7** (2.0, 6.7)	**<0.001**
Anxiety disorder	Yes	166 (35%)	69 (59%)	30 (79%)	**<0.001**	**2.2** (1.3, 3.8)	**<0.001**
Substance use disorder	Yes	94 (20%)	44 (38%)	18 (47%)	**<0.001**	**2.3** (1.3, 4.1)	**<0.001**

ARMHS: Australian Rural Mental Health Study; SD: Standard deviation; K10: Kessler-10 measure of psychological distress; AUDIT: Alcohol Use Disorder Identification Test; HOPES-12: Hunter Opinions and Personal Expectations Scale; SEIFA (IRSAD) Index of Relative Socio-Economic Advantage and Disadvantage; ASGC: Australian Standard Geographic Classification; AOR: Adjusted Odds Ratio.

^#^See the right-hand columns of Table 5 for the percentages of each predictor variable sub-category reporting lifetime suicidal ideation; the predictors were included in separate logistic regressions, controlling for age, gender, K10 psychological distress score, marital status and professional mental health service use (as appropriate); bolded p-values are statistically significant (against Bonferroni-adjusted thresholds).

The right-hand columns of Table 7 report similar logistic regression models to those presented earlier, but in this instance, each analysis was adjusted for the key factors that were significant in the parallel analyses (see Table 5): age, gender, psychological distress, marital status and professional mental health service use. Consequently, from the ARMHS sub-analysis we identified two potential additional predictors of lifetime suicidal ideation: lower optimism (HOPES-12; AOR 0.21, p < 0.001) and a (Bonferroni-adjusted non-significant) trend for lower infrastructure and service accessibility (AOR 0.91, p = 0.007). All lifetime psychiatric disorders also remained significant (p < 0.001) in these analyses (affective disorder AOR 3.7; anxiety disorder AOR 2.2; and substance use disorder AOR 2.3).

## Discussion

As suicide death rates have been consistently higher in rural than in urban settings, differences in rates of suicidal ideation or attempts and determinants of suicidal ideation and attempts across the geographical spectrum from urban to rural regions were explored. Any modifiable differences found could have a major impact on the development of interventions to prevent suicidal behaviour. Using the 2007-NSMHWB and ARMHS data in parallel analyses, we found no difference in the rates or the key determinants of suicidal ideation or attempts, across geographical region.

The main “determinants” of 12-month and lifetime suicidal ideation and lifetime suicide attempts across geographical regions were current psychological distress and psychiatric disorder. This is consistent with findings in previous studies of the general population [45, 46]. In our analyses, psychiatric disorder was associated with over a four-fold increase in the odds of reporting lifetime suicidal ideation in both datasets (see Table 5), with lifetime affective disorder making the largest contribution (AORs from 2.6 to 7.1). For lifetime suicide attempts the major contributors were reporting a lifetime affective disorder (with or without 12 month symptoms) or a lifetime anxiety disorder with 12 month symptoms (see Table 6). Geographical location was not significantly associated with suicidal ideation or attempts (see Table 3) and there were no associated interaction effects in the logistic regressions (see Additional file 3: Table S3, Additional file 4: Table S4 and Additional file 5: Table S5).

The consistently strong associations between current psychological distress and each of the outcomes (see Tables 4, 5, 6 and 7) may also partially reflect the influence of long-run symptomatology, as well as the ongoing disabling consequences that often accompany psychiatric conditions, such as employment disruption and/or associated financial adversity. It should be noted that within our analysis framework, current psychological distress was examined in relative isolation, whereas the contributions of psychiatric disorder, employment status, financial adversity and the other predictors were assessed with current psychological distress and the other key covariates statistically controlled.

The findings regarding psychiatric disorder and suicidal ideation and attempts strongly support the view that strategies to target prevention and early detection of psychiatric disorder are particularly relevant to suicide prevention [47]. The significant association between 12-month suicidal ideation and 12-month disorder rates could also be accounted for by overlap in symptom criteria for major depressive disorder, but not anxiety disorders. The link with 12-month anxiety disorder could be accounted for by comorbid depressive disorder in those with anxiety disorder. Previous analysis from this sample has indicated a significant independent effect of anxiety-related conditions, specifically post-traumatic stress disorder, on suicidal ideation and behaviours [48]. In addition, depression and suicide have been shown to be related but independent constructs [48, 49].

To better understand the rural–urban disparities in suicide requires research that goes beyond analysis of disorder rates alone and can explore the distribution of personal, social and community level factors that may have a bearing on the trajectory and impact of mental illness, and its personal and social impact [50, 51]. Important characteristics of non-urban areas that may impact health outcomes not well represented in existing geographic categories of ‘rurality’ or ‘remoteness’ [16, 17, 52] include district level socio-economic disadvantage and other characteristics of the populations themselves. Some examples include: indigeneity; higher proportions of the population working in occupations with attendant health risks (such as primary industries); lower access to specific health services; greater vulnerability to adverse environmental events, and greater vulnerability to adverse social and economic impacts when those events occur; vulnerability to population shifts; and geographic isolation.

In our analyses, social factors including employment, financial adversity and marital status were significantly associated with suicidal ideation and attempt and warrant further investigation. There is a large body of work linking unemployment with poorer mental health, including an earlier report from ARMHS indicating high levels of distress among rural unemployed [53]. Previous research has identified significant associations between financial hardship and incident major depression in an Australian sample [54]. It should also be noted that the presence in the current study of, for example, a modest but non-differential association (across regions) between financial hardship and lifetime suicidal ideation, tends to suggest that sub-regions with pervasive financial hardship (regardless of geographical location) will tend to experience higher rates of suicidality. However, the impact of personal financial hardship may be moderated by personal characteristics such as optimism and by resources of the district in which people reside, hence the importance of rural community characteristics. Previous analyses have indicated the significant role of social networks and community connection in mental health outcomes [55], consistent with a large body of work suggesting the moderating effects of social networks and support [56].

Other Australian research has also detected a significant association between loss of rural infrastructure, populations and resources, and population distress levels [57], and this is reflected in our findings regarding concern about rural infrastructure and services, and suicidal ideation. This may reflect the perceptions and experiences among those with high levels of distress and greater need for assistance, who become more conscious of the limitations of surrounding community resources.

The contexts across rural, regional and major cities are very different and it is feasible that psychological distress, suicidal ideation and mental disorder, in general, do not differ by geographical location; however death by suicide does [1]. One of the most effective methods of suicide prevention is restriction of access to lethal means [47]. The context in rural areas is very conducive to access to effective means of suicide, especially with access to and familiarity with firearms [17]. There is also access to isolation; hence it is easier not to be found in rural areas until it is too late, following potentially less lethal means such as hanging or overdose. There are also issues around help-seeking behaviours and rural culture [58, 59].

To better account for the differences in suicide rates between rural and metropolitan regions, above and beyond what we already know, further research is needed. Perhaps efforts would be better placed in evaluating targeted intervention programs for those at highest risk of death by suicide in rural areas including farmers and Indigenous peoples [60] and in developing evidenced based suicide prevention strategies.

### Strengths and limitations

Major strengths of this analysis are the use of a Composite International Diagnostic Interview in both samples to measure the outcomes and potential determinants at a similar point in time, the large sample size and the inclusion of a substantial proportion of participants from outer regional, remote and very remote regions. However, there are several limitations of this approach. Firstly, these two datasets provided coarse comparisons across regions, and were limited by the small number of common variables that assessed known determinants of suicidal ideation at a more fine-grained level (e.g., personality factors, social support, or valid measures of personal socio-economic factors). Both data sources provided cross-sectional data and therefore the temporal sequence between predictor (or exposure) variables and outcomes were unable to be determined. Despite this, these results have emphasised the importance of these factors. Secondly, we were unable to explore full geographical categories from urban to very remote, however, we did expand upon the dichotomous urban and rural approach by using three categories of remoteness. Thirdly, while the same outcome measures were used in both samples, the interview was delivered face-to-face for the 2007-NSMHWB and by telephone in ARHMS; this may have led to some differences that we were unable to account for in these analyses. However, telephone versus face-to-face has been examined previously using this instrument and no differences were found [61]. Fourthly, participants from the 2007-NSMHWB were randomly selected, while ARMHS participants in the current dataset were selected on a stratified basis from a random community sample with a low response rate (26% at baseline), however, appropriate statistical analyses have accounted for this selection strategy.

In addition, indigenous status was not asked as part of the 2007-NSMHWB and less than two percent of ARMHS participants stated they were of Aboriginal or Torres Strait Islander origin. In most states and territories in Australia, Aboriginal and Torres Strait Islander people have a suicide death rate twice that of non-indigenous Australians [62]. There may also be a survivor effect, where suicide attempts are more likely to be fatal in rural areas. Further to this, the quality and reliability of data collected on both suicide attempts and death by suicide becomes vital and is important to consider in areas where infrastructure and services may be limited, likely leading to an underestimation of death by suicide [63]. From the ARMHS sub-analysis availability of infrastructure and services and optimism were identified as two potential protective factors for suicidal ideation and require further exploration as part of future research in this area.

## Conclusions

The results of these analyses do not provide an explanation for why suicide death rates are higher in rural communities than in major cities. We were unable to show a difference between rates and determinants of suicidal ideation across geographical region. Given that rates of mental illness and rates of suicidal ideation are similar across geographical region, this suggests factors other than disorder are important in influencing rates of death from suicide in rural areas. The importance of prevention, and addressing and intervening in areas where there is a known difference, in particular access to lethal means and limited availability and access to mental health services, becomes increasingly important. So too does intervening in populations at increased risk of death by suicide such as Indigenous peoples and farmers.

## Electronic supplementary material

Additional file 1: **Detailed comparison of characteristics by region for 2007-NSMHWB and ARMHS samples.** (DOC 80 KB)

Additional file 2: **Suicide behaviour across regions for 2007-NSMHWB and ARMHS samples.** (DOC 62 KB)

Additional file 3: **Selected logistic regressions for 12-month suicidal ideation by remoteness for 2007-NSMHWB sample** ^**#**^ **.** Selected logistic regressions for 12-month suicidal ideation by remoteness for ARMHS sample^#^. (DOC 178 KB)

Additional file 4: **Selected logistic regressions for lifetime suicidal ideation by remoteness for 20007-NSMHWB sample** ^**#**^ **.** Selected logistic regressions for lifetime suicidal ideation by region for ARMHS sample^#^. (DOC 171 KB)

Additional file 5: **Selected logistic regressions for lifetime suicide attempts by remoteness for 20007-NSMHWB sample** ^**#**^ **.** Selected logistic regressions for lifetime suicide attempts by remoteness for ARMHS sample^#^. (DOC 178 KB)

## Abbreviations

ABS: Australian Bureau of Statistics
AOR: Adjusted Odds Ratio
ARMHS: Australian Rural Mental Health Study
AUDIT: Alcohol Use Disorder Identification Test
ASGC: Australian Standard Geographic Classification
CI: Confidence Interval
DSM-IV: Diagnostic and Statistical Manual – Fourth Edition
EPI: Eysenck Personality Inventory
HOPES-12: Hunter Opinions and Personal Expectations Scale
ICD-10: International Classification of Diseases – Tenth Edition
IOR: Interaction Odds Ratio
IRSAD: Index of Relative Socio-Economic Advantage and Disadvantage
NSMHWB: National Survey of Mental Health and Well-being
RAO1CX: ABS Remoteness variable
SD: Standard Deviation
WMH-CIDI-3.0: World Mental Health Composite International Diagnostic Interview version 3.0.
