# Supplementary material for: Determinants of suicidal ideation and suicide attempts: parallel cross-sectional analyses examining geographical location
Source: BMC Psychiatry. 2014 Jul 23;14:208. doi: 10.1186/1471-244X-14-208 (PMC4227072; doi:10.1186/1471-244X-14-208)
Supplement: Supplementary file 1 — Additional file 1: Detailed comparison of characteristics by region for 2007-NSMHWB and ARMHS samples. (DOC 80 KB) [file 12888_2014_1706_MOESM1_ESM.doc]

**Supplementary Table S1: Detailed comparison of characteristics by region for 2007-NSMHWB and ARMHS samples**

|  | | **2007-NSMHWB** | | | **ARMHS** | |
| --- | --- | --- | --- | --- | --- | --- |
| **Characteristic** | **Category** | **Major cities**  **n=5388**  **n (%)** | **Inner Regional**  **n=1943**  **n (%)** | **Other**  **n=1132**  **n (%)** | **Inner Regional**  **n=251**  **n (%)** | **Other**  **n=383**  **n (%)** |
| **Demographic factors** |  |  |  |  |  |  |
| Age | 18-44 | 2753 (51) | 785 (40) | 483 (43) | 60 (24) | 78 (20) |
|  | 45-64 | 1520 (28) | 660 (34) | 357 (32) | 135 (54) | 207 (54) |
|  | 65-85 | 1115 (21) | 498 (26) | 292 (26) | 56 (22) | 98 (26) |
| Gender | Male | 2436 (45) | 894 (46) | 513 (45) | 103 (41) | 147 (38) |
|  | Female | 2952 (55) | 1049 (54) | 619 (55) | 148 (59) | 236 (62) |
| Marital status | Not married | 2913 (54) | 945 (49) | 604 (53) | 93 (37) | 137 (36) |
|  | Married | 2475 (46) | 998 (51) | 528 (47) | 158 (63) | 243 (64) |
| Level of education | No university degree | 3245 (60) | 1413 (72) | 873 (77) | 185 (74) | 318 (83) |
|  | University or higher degree | 2143 (40) | 530 (27) | 259 (23) | 66 (26) | 65 (17) |
| Employment status | Employed | 3458 (64) | 1149 (59) | 670 (59) | 125 (50) | 208 (55) |
|  | Unemployed | 119 (2.2) | 33 (1.7) | 36 (3.2) | 9 (3.6) | 10 (2.6) |
|  | Not in workforce | 1811 (34) | 761 (39) | 426 (38) | 116 (46) | 161 (42) |
| Financial adversity | Low | 4657 (86) | 1667 (86) | 951 (84) | 143 (68) | 221 (69) |
|  | Moderate | 614 (11) | 224 (12) | 155 (14) | 48 (23) | 70 (22) |
|  | High | 117 (2.2) | 52 (2.7) | 26 (2.3) | 18 (8.6) | 27 (8.5) |
| **Physical health** |  |  |  |  |  |  |
| Smoking | No | 4271 (79) | 1499 (77) | 847 (75) | 176 (83) | 259 (81) |
|  | Yes | 1117 (21) | 444 (23) | 285 (25) | 37 (17) | 60 (19) |
| Number of chronic diseases | 0 | 3705 (69) | 1175 (60) | 710 (63) | 141 (56) | 216 (56) |
|  | 1 | 1201 (22) | 534 (27) | 294 (26) | 87 (35) | 121 (32) |
|  | >=2 | 482 (9.0) | 234 (12) | 128 (11) | 23 (9.2) | 46 (12) |
| **Mental Health** |  |  |  |  |  |  |
| Psychological distress (K10) | Low | 3824 (71) | 1380 (71) | 826 (73) | 70 (28) | 119 (31) |
|  | Moderate | 1232 (23) | 444 (23) | 249 (22) | 129 (51) | 190 (50) |
|  | High | 331 (6.0) | 118 (6.1) | 57 (5.0) | 52 (21) | 71 (19) |
| Any affective disorder | No lifetime diagnosis | 4581 (85) | 1660 (85) | 955 (84) | 186 (74) | 305 (80) |
|  | Lifetime diagnosis with 12 month symptoms | 322 (6.0) | 112 (5.8) | 68 (6.0) | 39 (16) | 38 (9.9) |
|  | Lifetime diagnosis with no 12 month symptoms | 485 (9.0) | 171 (8.8) | 109 9.6) | 26 (10) | 40 (10) |
| Any anxiety disorder | No lifetime diagnosis | 3988 (74) | 1432 (74) | 822 (73) | 137 (55) | 232 (61) |
|  | Lifetime diagnosis with 12 month symptoms | 748 (14) | 258 (13) | 162 (14) | 77 (31) | 88 (23) |
|  | Lifetime diagnosis with no 12 month symptoms | 652 (12) | 253 (13) | 148 (13) | 37 (15) | 63 (16) |
| Any substance use disorder | No lifetime diagnosis | 4164 (77) | 1453 (75) | 797 (70) | 193 (77) | 285 (74) |
|  | Lifetime diagnosis with 12 month symptoms | 253 (4.7) | 80 (4.1) | 52 (4.6) | 8 (3.2) | 11 (2.9) |
|  | Lifetime diagnosis with no 12 month symptoms | 971 (18) | 410 (21) | 283 (25) | 50 (20) | 87 (23) |
| Any lifetime psychiatric disorder | No | 2661 (49) | 875 (45) | 512 (45) | 88 (35) | 136 (36) |
|  | Yes | 2727 (51) | 1068 (55) | 620 (55) | 163 (65) | 247 (64) |
| Any 12-month psychiatric disorder | No | 4158 (77) | 1494 (77) | 873 (77) | 152 (61) | 242 (63) |
|  | Yes | 1230 (23) | 449 (23) | 259 (23) | 99 (39) | 141 (37) |
| Two or more psychiatric disorders | No | 3957 (73) | 1415 (73) | 816 (72) | 140 (56) | 240 (63) |
|  | Yes | 1431 (27) | 528 (27) | 316 (28) | 111 (44) | 143 (37) |
| **Health service use** |  |  |  |  |  |  |
| Any professional mental health service use | No | 4582 (85) | 1682 (87) | 992 (88) | 186 (74) | 288 (75) |
|  | Yes | 806 (15) | 261 (13) | 140 (12) | 65 (26) | 95 (25) |
| Consulted a mental health professional in last 12 months and did not get enough help/info as needed | No, needs met | 348 (81) | 106 (86) | 53 (82) | 43 (62) | 62 (63) |
|  | Yes, unmet need | 84 (19) | 17 (14) | 12 (18) | 26 (38) | 37 (37) |
