# Supplementary material for: Determinants of suicidal ideation and suicide attempts: parallel cross-sectional analyses examining geographical location
Source: BMC Psychiatry. 2014 Jul 23;14:208. doi: 10.1186/1471-244X-14-208 (PMC4227072; doi:10.1186/1471-244X-14-208)
Supplement: Supplementary file 2 — Additional file 2: Suicide behaviour across regions for 2007-NSMHWB and ARMHS samples. (DOC 62 KB) [file 12888_2014_1706_MOESM2_ESM.doc]

**Supplementary Table S2: Suicide behaviour across regions for 2007-NSMHWB and ARMHS samples**

|  | | **2007-NSMHWB** | | | **ARMHS** | |
| --- | --- | --- | --- | --- | --- | --- |
| **Question** | **Category** | **Major cities**  **n=5388**  **n (%)** | **Inner Regional**  **n=1943**  **n (%)** | **Other**  **n=1132**  **n (%)** | **Inner Regional**  **n=251**  **n (%)** | **Other**  **n=383**  **n (%)** |
| Number of different sad/discouraged/uninterested episodes | n | 966 | 352 | 197 | 74 | 102 |
|  | mean (SD) | 5 (9) | 4 (9) | 5 (9) | 6 (8) | 7 (10) |
|  | median (min, max) | 2 (1, 80) | 2 (1,70) | 2 (1, 80) | 4 (1, 50) | 3 (1, 50) |
| Age first seriously thought about committing suicide | n | 747 | 287 | 176 | 60 | 83 |
|  | mean (SD) | 28 (14) | 31 (15) | 30 (15) | 28 (16) | 33 (16) |
|  | median (min, max) | 23 (5, 82) | 27 (8, 78) | 26 (8, 78) | 23 (7, 70) | 30 (5, 73) |
| Age last seriously thought about committing suicide | n | 751 | 288 | 175 | 44 | 69 |
|  | mean (SD) | 33 (14) | 35 (16) | 34 (15) | 36 (14) | 40 (14) |
|  | median (min, max) | 30 (7, 83) | 34 (12, 78) | 32 (12, 78) | 37 (11, 66) | 41 (14, 73) |
| Number of suicide attempts | n | 192 | 71 | 51 | 19 | 27 |
|  | mean (SD) | 2 (1) | 2 (1) | 2 (1) | 2 (1) | 3 (3) |
|  | median (min, max) | 1 (1, 5) | 1 (1, 5) | 1 (1, 5) | 1 (1, 6) | 2 (1, 18) |
| Age in years at first suicide attempt | n | 192 | 71 | 53 | 8 | 14 |
|  | mean (SD) | 23 (11) | 27 (13) | 28 (13) | 26 (14) | 18 (6) |
|  | median | 20 | 23 | 25 | 20 | 17 |
| Result of first suicide attempt | I made a serious attempt to kill myself and it was only luck that I did not succeed. | 48 (56%) | 22 (71%) | 13 (65%) | 1 (11%) | 8 (57%) |
|  | I tried to kill myself, but knew that the method was not foolproof. | 13 (15%) | 4 (13%) |  | 1 (11%) | 3 (21%) |
|  | My attempt was a cry for help. I did not intend to die. | 23 (27%) | 5 (16%) | 6 (30%) | 5 (56%) | 3 (21%) |
|  | Don t know | 2 (2.3%) |  | 1 (5.0%) | 2 (22%) |  |
| Age at last suicide attempt | n | 192 | 70 | 52 | 17 | 23 |
|  | mean (SD) | 26 (11) | 30 (12) | 30 (12) | 28 (12) | 32 (14) |
|  | median | 24 | 31 | 28 | 28 | 28 |
| Suicide attempt in past 12 months resulted in injury/poisoning | Yes | 13 (65%) | 5 (83%) | 4 (44%) | 2 (67%) | 2 (67%) |
|  |  |  |  |  |  |  |
| Suicide attempt in past 12 months required medical attention | Yes | 8 (62%) | 2 (40%) | 3 (75%) | 1 (50%) | 2 (100%) |
|  |  |  |  |  |  |  |
| Suicide attempt in past 12 months required overnight hospitalisation | Yes | 6 (75%) | 2 (100%) | 3 (100%) |  | 1 (50%) |
|  |  |  |  |  |  |  |
| Result of last suicide attempt | I made a serious attempt to kill myself and it was only luck that I did not succeed. | 46 (53%) | 21 (68%) | 8 (40%) | 7 (37%) | 10 (37%) |
|  | I tried to kill myself, but knew that the method was not foolproof | 8 (9.3%) | 3 (9.7%) | 2 (10%) | 5 (26%) | 5 (19%) |
|  | My attempt was a cry for help. I did not intend to die. | 30 (35%) | 7 (23%) | 10 (50%) | 7 (37%) | 12 (44%) |
|  | Don’t know | 2 (2.3%) |  |  |  |  |

NSMHWB: National Survey of Mental Health and Well-being (aged 18-85), ARMHS: Australian Rural Mental Health Study, unweighted sample (aged 18-85) who completed the WMH-CIDI-3.0 component.
