# Supplementary material for: Determinants of suicidal ideation and suicide attempts: parallel cross-sectional analyses examining geographical location
Source: BMC Psychiatry. 2014 Jul 23;14:208. doi: 10.1186/1471-244X-14-208 (PMC4227072; doi:10.1186/1471-244X-14-208)
Supplement: Supplementary file 3 — Additional file 3: Selected logistic regressions for 12-month suicidal ideation by remoteness for 2007-NSMHWB sample # . Selected logistic regressions for 12-month suicidal ideation by remoteness for ARMHS sample#. (DOC 178 KB) [file 12888_2014_1706_MOESM3_ESM.doc]

**Supplementary Table S3a – Selected logistic regressions for 12-month suicidal ideation by remoteness** for 2007-NSMHWB sample#

| **Characteristic**  **(predictor or exposure variable)** | **Category** | **Major cities (n=5388) n (%)** | **Inner Regional (n=1943) n (%)** | **Other (n=1132) n (%)** | **AOR** | **99% CI** | **p-value** | **IOR** | **p-value** |
| --- | --- | --- | --- | --- | --- | --- | --- | --- | --- |
| **Demographic factors** |  |  |  |  |  |  |  |  |  |
| Age in years | 18-44 | 93 (3.4) | 20 (2.5) | 15 (3.1) | . | . | . | . | . |
|  | 45-64 | 41 (2.7) | 19 (2.9) | 10 (2.8) | 0.87 | (0.58, 1.3) | 0.381 | 1.0 | 0.925 |
|  | 65-85 | 11 (1.0) | 9 (1.8) | 4 (1.4) | 0.58 | (0.32, 1.1) | 0.023 | 1.2 | 0.571 |
| Gender | Male | 53 (2.2) | 24 (2.7) | 14 (2.7) | . | . | . | . | . |
|  | Female | 92 (3.1) | 24 (2.3) | 15 (2.4) | 0.93 | (0.64, 1.4) | 0.606 | 0.70 | 0.085 |
| Marital status | Not married | 116 (4.0) | 34 (3.6) | 25 (4.1) | . | . | . | . | . |
|  | Married | 29 (1.2) | 14 (1.4) | 4 (0.8) | **0.43** | **(0.28, 0.67)** | **<0.001** | 0.89 | 0.630 |
| Level of education | No university degree | 92 (2.8) | 38 (2.7) | 18 (2.1) | . | . | . | . | . |
|  | University or higher degree | 53 (2.5) | 10 (1.9) | 11 (4.2) | 1.0 | (0.68, 1.5) | 0.969 | 1.2 | 0.370 |
| Employment status | Employed | 78 (2.3) | 21 (1.8) | 15 (2.2) | . | . | . | . | . |
|  | Not in Workforce | 7 (5.9) | 1 (3.0) | 3 (8.3) | 1.5 | (1.0, 2.3) | 0.011 | 0.96 | 0.857 |
|  | Unemployed | 60 (3.3) | 26 (3.4) | 11 (2.6) | 1.8 | (0.73, 4.3) | 0.095 | 1.1 | 0.830 |
| Financial adversity | Low | 88 (1.9) | 26 (1.6) | 12 (1.3) | . | . | . | . | . |
|  | Medium | 38 (6.2) | 14 (6.3) | 12 (7.7) | **1.7** | **(1.1, 2.7)** | **0.001** | 1.4 | 0.144 |
|  | High | 19 (16) | 8 (15) | 5 (19) | **2.9** | **(1.6, 5.4)** | **<0.001** | 1.4 | 0.283 |
| **Physical health** |  |  |  |  |  |  |  |  |  |
| Smoking | No | 85 (2.0) | 31 (2.1) | 18 (2.1) | . | . | . | . | . |
|  | Yes | 60 (5.4) | 17 (3.8) | 11 (3.9) | 1.4 | (0.93, 2.1) | 0.033 | 0.81 | 0.306 |
| Number of chronic diseases | 0 | 101 (2.7) | 26 (2.2) | 22 (3.1) | . | . | . | . | . |
|  | 1 | 27 (2.2) | 14 (2.6) | 3 (1.0) | 0.85 | (0.52, 1.4) | 0.391 | 0.75 | 0.279 |
|  | >=2 | 17 (3.5) | 8 (3.4) | 4 (3.1) | 1.4 | (0.75, 2.6) | 0.161 | 0.93 | 0.810 |
| **Mental health** |  |  |  |  |  |  |  |  |  |
| Psychological distress (K10) | Low | 20 (0.5) | 3 (0.2) | 5 (0.6) | . | . | . | . | . |
|  | Moderate | 59 (4.8) | 18 (4.1) | 13 (5.2) | **9.7** | **(5.6, 17)** | **<0.001** | 0.98 | 0.937 |
|  | High | 66 (20) | 26 (22) | 10 (18) | **50** | **(29, 88)** | **<0.001** | 0.97 | 0.923 |
| Any affective disorder | No lifetime diagnosis | 70 (1.5) | 22 (1.3) | 11 (1.2) | **.** | **.** | **.** | . | . |
|  | Lifetime diagnosis with 12 month symptoms | 63 (20) | 21 (19) | 14 (21) | **3.9** | **(2.5, 6.1)** | **<0.001** | 1.2 | 0.345 |
|  | Lifetime diagnosis with no 12 month symptoms | 12 (2.5) | 5 (2.9) | 4 (3.7) | 0.97 | (0.51, 1.8) | 0.895 | 1.5 | 0.218 |
| Any anxiety disorder | No lifetime diagnosis | 58 (1.5) | 20 (1.4) | 11 (1.3) | . | . | . | . | . |
|  | Lifetime diagnosis with 12 month symptoms | 75 (10) | 23 (9.0) | 18 (11) | **2.0** | **(1.3, 3.1)** | **<0.001** | 1.1 | 0.596 |
|  | Lifetime diagnosis with no 12 month symptoms | 12 (1.8) | 5 (2.0) | 0 (0.0) | 0.62 | (0.31, 1.3) | 0.083 | 0.60 | 0.253 |
| Any substance use disorders | No lifetime diagnosis | 88 (2.1) | 24 (1.7) | 15 (1.9) | . | . | . | . | . |
|  | Lifetime diagnosis with 12 month symptoms | 29 (11) | 9 (11) | 7 (13) | **2.9** | **(1.7, 5.0)** | **<0.001** | 1.3 | 0.323 |
|  | Lifetime diagnosis with no 12 month symptoms | 28 (2.9) | 15 (3.7) | 7 (2.5) | 1.1 | (0.68, 1.8) | 0.623 | 1.0 | 0.962 |
| Any lifetime psychiatric disorder | No | 24 (0.9) | 4 (0.5) | 3 (0.6) | . | . | . | . | . |
|  | Yes | 121 (4.4) | 44 (4.1) | 26 (4.2) | **2.0** | **(1.2, 3.5)** | **0.001** | 1.4 | 0.303 |
| Any 12 month psychiatric disorder | No | 38 (0.9) | 11 (0.7) | 7 (0.8) | **.** | **.** | **.** | . | . |
|  | Yes | 107 (8.7) | 37 (8.2) | 22 (8.5) | **3.5** | **(2.2, 5.5)** | **<0.001** | 1.2 | 0.428 |
| Two or more psychiatric disorders | No | 50 (1.3) | 14 (1.0) | 9 (1.1) | **.** | **.** | **.** | . | . |
|  | Yes | 95 (6.6) | 34 (6.4) | 20 (6.3) | **1.9** | **(1.2, 2.9)** | **0.001** | 1.1 | 0.613 |
| **Health service use** |  |  |  |  |  |  |  |  |  |
| Any professional mental health service use in past 12 months | No | 57 (1.2) | 15 (0.9) | 12 (1.2) | . | . | . | . | . |
|  | Yes | 88 (11) | 33 (13) | 17 (12) | **4.1** | **(2.7, 6.2)** | **<0.001** | 1.1 | 0.580 |
| Consulted a mental health professional in last 12 months and did not get enough help/info as needed | No, needs met | 41 (12) | 16 (15) | 8 (15) | . | . | . | . | . |
|  | Yes, unmet need | 24 (29) | 6 (35) | 2 (17) | 1.8 | (0.92, 3.6) | 0.023 | 0.77 | 0.516 |

NSMHWB: National Survey of Mental Health and Well-being (aged 18-85).

# Bracketed values refer to the percentage of each predictor variable sub-category reporting 12-month suicidal ideation; see supplementary Table S1 for cell sizes.

Note: Each predictor variable was included in a separate logistic regression, controlling for age, gender, and K10 psychological distress score (as appropriate); AOR: Adjusted Odds Ratio - adjusted for the covariates; IOR: Interaction Odds Ratio, testing Predictor variable x Region interaction; bolded p-values are statistically significant (against Bonferroni-adjusted thresholds).

**Supplementary Table S3b: Selected logistic regressions for 12-month suicidal ideation by remoteness for ARMHS sample^#^**

| **Characteristic**  **(predictor or exposure variable)** | **Category** | **Inner Regional**  **(n=251)**  **n (%)** | **Other**  **(n=383) n (%)** | **AOR** | **(99% CI)** | **p-value** | **IOR** | **p-value** |
| --- | --- | --- | --- | --- | --- | --- | --- | --- |
| **Demographic factors** |  |  |  |  |  |  |  |  |
| Age in years | 18-44 | 9 (15) | 5 (6.4) |  |  | . | . | . |
|  | 45-64 | 11 (8.1) | 8 (3.9) | 0.57 | (0.21, 1.5) | 0.143 | 1.0 | 0.982 |
|  | 65-85 | 2 (3.6) | 3 (3.0) | 0.40 | (0.10, 1.7) | 0.105 | 0.74 | 0.792 |
| Gender | Male | 10 (9.7) | 6 (4.1) |  |  | . | . | . |
|  | Female | 12 (8.1) | 10 (4.2) | 0.83 | (0.33, 2.1) | 0.596 | 0.83 | 0.802 |
| Marital status | Not married | 12 (13) | 9 (6.6) |  |  | . | . | . |
|  | Currently married | 10 (6.3) | 7 (2.9) | 0.65 | (0.26, 1.6) | 0.218 | 0.96 | 0.954 |
| Level of education | No university degree | 17 (9.2) | 12 (3.8) |  |  | . | . | . |
|  | University of higher degree | 5 (7.6) | 4 (6.2) | 1.1 | (0.38, 3.2) | 0.805 | 0.60 | 0.540 |
| Employment status | Employed | 9 (7.2) | 9 (4.3) |  |  | . | . | . |
|  | Not in workforce | 10 (8.6) | 7 (4.3) | 1.6 | (0.56, 4.4) | 0.249 | 1.19 | 0.810 |
|  | Unemployed | 3 (33) |  | 2.0 | (0.31, 12) | 0.338 | 9E5 | 0.983 |
| Financial adversity | Low | 7 (4.9) | 7 (3.2) |  |  | . | . | . |
|  | Medium | 7 (15) | 4 (5.7) | 1.7 | (0.55, 5.4) | 0.217 | 1.62 | 0.590 |
|  | High | 3 (17) | 3 (11) | 1.3 | (0.31, 5.7) | 0.601 | 0.77 | 0.806 |
| **Physical health** |  |  |  |  |  |  |  |  |
| Smoking | No | 15 (8.5) | 8 (3.1) |  |  | . | . | . |
|  | Yes | 3 (8.1) | 7 (12) | 1.1 | (0.37, 3.4) | 0.794 | 0.24 | 0.106 |
| Number of chronic diseases | 0 | 13 (9.2) | 10 (4.6) |  |  | . | . | . |
|  | 1 | 6 (6.9) | 5 (4.1) | 1.0 | (0.34, 3.0) | 0.988 | 0.69 | 0.639 |
|  | >=2 | 3 (13) | 1 (2.2) | 1.2 | (0.21, 6.7) | 0.787 | 4.09 | 0.279 |
| **Mental health** |  |  |  |  |  |  |  |  |
| Psychological distress | Low | 1 (1.4) |  |  |  | . | . | . |
|  | Moderate | 7 (5.4) | 8 (4.2) | 8.9 | (0.61, 13) | 0.036 | 0.00 | 0.956 |
|  | High | 14 (27) | 8 (11) | **36** | **(2.5, 518)** | **<0.001** | 0.00 | 0.960 |
| Any affective disorder | No lifetime diagnosis | 7 (3.8) | 6 (2.0) |  |  | **.** | . | . |
|  | Lifetime diagnosis with 12 month symptoms | 13 (33) | 6 (16) | **5.5** | **(1.7, 18)** | **<0.001** | 1.3 | 0.732 |
|  | Lifetime diagnosis with no 12 month symptoms | 2 (7.7) | 4 (10) | 2.7 | (0.70, 11) | 0.057 | 0.41 | 0.410 |
| Any anxiety disorder | No lifetime diagnosis | 5 (3.6) | 3 (1.3) |  |  | . | . | . |
|  | Lifetime diagnosis with 12 month symptoms | 16 (21) | 8 (9.1) | **4.3** | **(1.4, 14)** | **0.001** | 0.86 | 0.866 |
|  | Lifetime diagnosis with no 12 month symptoms | 1 (2.7) | 5 (7.9) | 2.4 | (0.56, 10) | 0.121 | 0.10 | 0.100 |
| Any substance use disorders | No lifetime diagnosis | 13 (6.7) | 7 (2.5) |  |  | . | . | . |
|  | Lifetime diagnosis with 12 month symptoms | 4 (50) | 5 (45) | **11** | **(2.4, 50)** | **<0.001** | 0.68 | 0.750 |
|  | Lifetime diagnosis with no 12 month symptoms | 5 (10) | 4 (4.6) | 1.1 | (0.34, 3.3) | 0.898 | 1.0 | 0.972 |
| Any lifetime psychiatric disorder | No |  | 1 (0.7) |  |  | . | . | . |
|  | Yes | 22 (13) | 15 (6.1) | 11 | (0.76, 158) | 0.020 | 51E3 | 0.944 |
| Any 12 month psychiatric disorder | No | 4 (2.6) | 1 (0.4) |  |  | . | . | . |
|  | Yes | 18 (18) | 15 (11) | **6.8** | **(1.8, 26)** | **<0.001** | 0.34 | 0.372 |
| Two or more psychiatric disorders | No | 3 (2.1) | 4 (1.6) |  |  | . | . | . |
|  | Yes | 19 (18) | 12 (8.6) | **4.3** | **(1.3, 14)** | **0.001** | 2.11 | 0.397 |
| **Health service use** |  |  |  |  |  |  |  |  |
| Any professional mental health service use | No | 10 (5.5) | 7 (2.5) |  |  | . | . | . |
|  | Yes | 12 (17) | 9 (8.7) | 2.0 | (0.80, 5.2) | 0.048 | 0.82 | 0.783 |
| Consulted a mental health professional in last 12months and did not get as much help/info as needed | No | 6 (13) | 4 (5.6) |  |  | . | . | . |
|  | Yes | 6 (23) | 6 (16) | 2.0 | (0.56, 6.9) | 0.162 | 0.50 | 0.482 |

ARMHS: Australian Rural Mental Health Study, unweighted sample (aged 18-85) who completed the CIDI component.

# Bracketed values refer to the percentage of each predictor variable sub-category reporting 12-month suicidal ideation; see supplementary Table S1 for cell sizes.

Note: Each predictor variable was included in a separate logistic regression, controlling for age, gender, and K10 psychological distress score (as appropriate); AOR: Adjusted Odds Ratio - adjusted for the covariates; IOR: Interaction Odds Ratio, testing Predictor variable x Region interaction; bolded p-values are statistically significant (against Bonferroni-adjusted thresholds)..
