# Supplementary material for: Determinants of suicidal ideation and suicide attempts: parallel cross-sectional analyses examining geographical location
Source: BMC Psychiatry. 2014 Jul 23;14:208. doi: 10.1186/1471-244X-14-208 (PMC4227072; doi:10.1186/1471-244X-14-208)
Supplement: Supplementary file 4 — Additional file 4: Selected logistic regressions for lifetime suicidal ideation by remoteness for 20007-NSMHWB sample # . Selected logistic regressions for lifetime suicidal ideation by region for ARMHS sample#. (DOC 171 KB) [file 12888_2014_1706_MOESM4_ESM.doc]

**Supplementary Table S4a – Selected logistic regressions for lifetime suicidal ideation by remoteness for 20007-NSMHWB sample^#^**

| **Characteristic**  **(predictor or exposure**  **variable)** | **Category** | **Major cities (n=5388) n (5%)** | **Inner Regional (n=1943) n (%)** | **Other (n=1132)**  **n (%)** | **AOR** | **99% CI** | **p-value** | **IOR** | **p-value** |
| --- | --- | --- | --- | --- | --- | --- | --- | --- | --- |
| **Demographic factors** |  |  |  |  |  |  |  |  |  |
| Age in years | 18-44 | 401 (15) | 121 (15) | 91 (19) | . | . | . | . | . |
|  | 45-64 | 268 (18) | 128 (19) | 66 (18) | **1.3** | **(1.1, 1.6)** | **<0.001** | 0.84 | 0.075 |
|  | 65-85 | 87 (7.8) | 40 (8.0) | 22 (7.5) | **0.59** | **(0.45, 0.76)** | **<0.001** | 0.78 | 0.067 |
| Gender | Male | 310 (13) | 114 (13) | 79 (15) | . | . | . | . | . |
|  | Female | 446 (15) | 175 (17) | 100 (16) | 1.1 | (0.91, 1.3) | 0.254 | 0.96 | 0.631 |
| Marital status | Not married | 529 (18) | 185 (20) | 120 (20) | . | . | . | . | . |
|  | Married | 227 (9.2) | 104 (10) | 59 (11) | **0.54** | **(0.45, 0.65)** | **<0.001** | 1.0 | 0.783 |
| Level of education | No university degree | 471 (15) | 221 (16) | 130 (15) | . | . | . | . | . |
|  | University degree or higher | 285 (13) | 68 (13) | 49 (19) | 0.87 | (0.73, 1.1) | 0.056 | 1.0 | 0.743 |
| Employment Status | Employed | 480 (14) | 164 (14) | 97 (14) | . | . | . | . | . |
|  | Not in Workforce | 32 (27) | 6 (18) | 8 (22) | 1.3 | (1.0, 1.6) | 0.004 | 1.18 | 0.087 |
|  | Unemployed | 244 (13) | 119 (16) | 74 (17) | 1.5 | (0.95, 2.5) | 0.021 | 0.77 | 0.277 |
| Financial adversity | Low | 536 (12) | 210 (13) | 121 (13) | . | . | . | . | . |
|  | Medium | 157 (26) | 56 (25) | 48 (31) | **1.7** | **(1.3, 2.1)** | **<0.001** | 1.0 | 0.761 |
|  | High | 63 (54) | 23 (44) | 10 (38) | **3.5** | **(2.2, 5.1)** | **<0.001** | 0.66 | 0.059 |
| **Physical health** |  |  |  |  |  |  |  |  |  |
| Smoking | No | 507 (12) | 181 (12) | 104 (12) | . | . | . | . | . |
|  | Yes | 249 (22) | 108 (24) | 75 (26) | **1.8** | **(1.5, 2.1)** | **<0.001** | 1.2 | 0.142 |
| Number of chronic diseases | 0 | 492 (13) | 159 (14) | 109 (15) | . | . | . | . | . |
|  | 1 | 177 (15) | 82 (15) | 46 (16) | 1.3 | (1.0, 1.6) | 0.004 | 0.94 | 0.584 |
|  | >=2 | 87 (18) | 48 (21) | 24 (19) | **2.0** | **(1.5, 2.6)** | **<0.001** | 0.94 | 0.646 |
| **Mental Health** |  |  |  |  |  |  |  |  |  |
| Psychological distress (K10) | Low | 308 (8.1) | 115 (8.3) | 85 (10) | . | . | . | . | . |
|  | Moderate | 282 (23) | 110 (25) | 64 (26) | **3.3** | **(2.7, 3.9)** | **<0.001** | 0.957 | 0.653 |
|  | High | 166 (50) | 63 (53) | 30 (53) | **11** | **(8.2, 13)** | **<0.001** | 0.956 | 0.755 |
| Any affective disorder | No lifetime diagnosis | 397 (8.7) | 158 (9.5) | 95 (9.9) | **.** | **.** | **.** | . | . |
|  | Lifetime diagnosis with 12 month symptoms | 157 (49) | 59 (53) | 31 (46) | **4.2** | **(3.2, 5.6)** | **<0.001** | 0.91 | 0.517 |
|  | Lifetime diagnosis with no 12 month symptoms | 202 (42) | 72 (42) | 53 (49) | **5.8** | **(4.6, 7.3)** | **<0.001** | 1.1 | 0.602 |
| Any anxiety disorder | No lifetime diagnosis | 308 (7.7) | 125 (8.7) | 76 (9.2) | **.** | **.** | **.** | . | . |
|  | Lifetime diagnosis with 12 month symptoms | 278 (37) | 100 (39) | 61 (38) | **3.6** | **(2.9, 4.6)** | **<0.001** | 0.94 | 0.593 |
|  | Lifetime diagnosis with no 12 month symptoms | 170 (26) | 64 (25) | 42 (28) | **3.2** | **(2.6, 4.0)** | **<0.001** | 0.92 | 0.456 |
| Any substance use disorders | No lifetime diagnosis | 438 (11) | 170 (12) | 96 (12) | **.** | **.** | **.** | . | . |
|  | Lifetime diagnosis with 12 month symptoms | 86 (34) | 23 (29) | 13 (25) | **2.6** | **(1.9, 3.7)** | **<0.001** | 0.77 | 0.144 |
|  | Lifetime diagnosis with no 12 month symptoms | 232 (24) | 96 (23) | 70 (25) | **2.5** | **(2.0, 3.0)** | **<0.001** | 0.890 | 0.241 |
| Any lifetime psychiatric disorders | No | 107 (4.0) | 38 (4.3) | 28 (5.5) | **.** | **.** | **.** | . | . |
|  | Yes | 649 (24) | 251 (24) | 151 (24) | **4.7** | **(3.7, 5.9)** | **<0.001** | 0.88 | 0.285 |
| Any 12 month psychiatric disorder | No | 344 (8.3) | 143 (9.6) | 98 (11) | **.** | **.** | **.** | . | . |
|  | Yes | 412 (33) | 146 (33) | 81 (31) | **2.8** | **(2.3, 3.4)** | **<0.001** | 0.83 | 0.051 |
| Two or more psychiatric disorders | No | 262 (6.6) | 109 (7.7) | 63 (7.7) | **.** | **.** | **.** | . | . |
|  | Yes | 494 (35) | 180 (34) | 116 (37) | **4.6** | **(3.8, 5.6)** | **<0.001** | 0.948 | 0.566 |
| **Health service use** |  |  |  |  |  |  |  |  |  |
| Any professional mental health service use | No | 445 (9.7) | 173 (10) | 115 (12) | **.** | **.** | **.** | . | . |
|  | Yes | 311 (39) | 116 (44) | 64 (46) | **3.6** | **(3.0, 4.4)** | **<0.001** | 1.024 | 0.820 |
| Consulted a mental health professional in last 12 months and did not get as much help/info as needed | No, needs met | 120 (34) | 46 (43) | 21 (40) | . | . | . | . | . |
|  | Yes, unmet need | 50 (60) | 10 (59) | 6 (50) | 1.7 | (0.95, 3.0) | 0.019 | 0.75 | 0.379 |

NSMHWB: National Survey of Mental Health and Well-being (aged 18-85).

# Bracketed values refer to the percentage of each predictor variable sub-category reporting lifetime suicidal ideation; see supplementary Table S1 for cell sizes.

Note: Each predictor variable was included in a separate logistic regression, controlling for age, gender, and K10 psychological distress score (as appropriate); AOR: Adjusted Odds Ratio - adjusted for the covariates; IOR: Interaction Odds Ratio, testing Predictor variable x Region interaction; bolded p-values are statistically significant (against Bonferroni-adjusted thresholds).

**Supplementary Table S4b – Selected logistic regressions for lifetime suicidal ideation by region for ARMHS sample^#^**

| **Characteristic**  **(predictor or exposure variable)e** | **Category** | **Inner Regional (n=251)**  **n (%)** | **Other (n=383)**  **n (%)** | **AOR** | **p-value** | **IOR** | **p-value** |
| --- | --- | --- | --- | --- | --- | --- | --- |
| **Demographic factors** |  |  |  |  |  |  |  |
| Age in years | 18-44 | 25 (42) | 22 (28) |  | . | . | . |
|  | 45-64 | 36 (27) | 48 (23) | 0.68 | 0.095 | 0.76 | 0.552 |
|  | 65-85 | 5 (8.9) | 18 (18) | 0.43 | 0.005 | 0.32 | 0.088 |
| Gender | Male | 27 (26) | 26 (18) |  | . | . | . |
|  | Female | 39 (26) | 62 (26) | 1.2 | 0.283 | 0.62 | 0.245 |
| Married | Not married | 31 (33) | 51 (37) |  | . | . | . |
|  | Currently married | 35 (22) | 36 (15) | **0.48** | **<0.001** | 2.0 | 0.085 |
| University or higher degree | No university degree | 49 (26) | 72 (23) |  | . | . | . |
|  | University degree or higher | 17 (26) | 16 (25) | 0.94 | 0.784 | 0.93 | 0.873 |
| Employment status | Employed | 30 (24) | 44 (21) |  | . | . | . |
|  | Not in Workforce | 30 (26) | 42 (26) | 1.9 | 0.006 | 0.73 | 0.449 |
|  | Unemployed | 5 (56) | 2 (20) | 1.4 | 0.492 | 3.4 | 0.275 |
| Financial adversity | 0 | 25 (17) | 41 (19) |  | . | . | . |
|  | 1-2 | 22 (46) | 20 (29) | 1.9 | 0.009 | 2.6 | 0.064 |
|  | >=3 | 6 (33) | 13 (48) | 1.6 | 0.223 | 0.42 | 0.243 |
| **Physical health** |  |  |  |  |  |  |  |
| Smoking | No | 43 (24) | 53 (20) |  | . | . | . |
|  | Yes | 12 (32) | 22 (37) | 1.2 | 0.444 | 0.71 | 0.528 |
| Number of chronic diseases | 0 | 43 (30) | 60 (28) |  | . | . | . |
|  | 1 | 18 (21) | 21 (17) | 0.63 | 0.055 | 1.0 | 0.925 |
|  | >=2 | 5 (22) | 7 (15) | 0.58 | 0.164 | 1.6 | 0.490 |
| **Mental health** |  |  |  |  |  |  |  |
| Psychological distress (K10) | Low | 9 (13) | 9 (7.6) |  | . | . | . |
|  | Moderate | 30 (23) | 46 (24) | **2.7** | **<0.001** | 0.48 | 0.202 |
|  | High | 27 (52) | 33 (45) | **7.9** | **<0.001** | 0.65 | 0.486 |
| Any affective disorder | No lifetime diagnosis | 29 (16) | 47 (15) |  | **.** | . | . |
|  | Lifetime diagnosis with 12 month symptoms | 23 (59) | 16 (42) | **2.6** | **0.001** | 2.1 | 0.185 |
|  | Lifetime diagnosis with no 12 month symptoms | 14 (54) | 25 (63) | **7.1** | **<0.001** | 0.79 | 0.699 |
| Any anxiety disorder | No lifetime diagnosis | 19 (14) | 36 (16) |  | **.** | . | . |
|  | Lifetime diagnosis with 12 month symptoms | 37 (48) | 33 (38) | **2.7** | **<0.001** | 1.8 | 0.208 |
|  | Lifetime diagnosis with no 12 month symptoms | 10 (27) | 19 (30) | 2.1 | 0.009 | 1.0 | 0.941 |
| Any substance use disorder | No lifetime diagnosis | 39 (20) | 53 (19) |  | . | . | . |
|  | Lifetime diagnosis with 12 month symptoms | 6 (75) | 7 (64) | **6.7** | **<0.001** | 2.4 | 0.463 |
|  | Lifetime diagnosis with no 12 month symptoms | 21 (42) | 28 (32) | **2.0** | **0.003** | 1.7 | 0.240 |
| Any lifetime psychiatric disorder | No | 4 (4.5) | 11 (8.1) |  | **.** | . | . |
|  | Yes | 62 (38) | 77 (31) | **4.7** | **<0.001** | 3.0 | 0.091 |
| Any 12 month psychiatric disorder | No | 22 (14) | 36 (15) |  | . | . | . |
|  | Yes | 44 (44) | 52 (37) | **2.3** | **<0.001** | 1.8 | 0.161 |
| Two or more psychiatric disorders | No | 17 (12) | 34 (14) |  | **.** | . | . |
|  | Yes | 49 (45) | 54 (39) | **3.2** | **<0.001** | 1.8 | 0.156 |
| **Health service use** |  |  |  |  |  |  |  |
| Any professional mental health service use | No | 37 (20) | 47 (17) |  | **.** | . | . |
|  | Yes | 29 (41) | 41 (39) | **2.1** | **<0.001** | 0.80 | 0.600 |
| Consulted a mental health professional in last 12months and did not get as much help/info as needed | No, needs met | 17 (35) | 27 (38) |  | . | . | . |
|  | Yes, unmet needs | 14 (54) | 18 (49) | 1.4 | 0.291 | 1.2 | 0.827 |

ARMHS: Australian Rural Mental Health Study, unweighted sample (aged 18-85) who completed the CIDI component.

# Bracketed values refer to the percentage of each predictor variable sub-category reporting lifetime suicidal ideation; see supplementary Table S1 for cell sizes.

Note: Each predictor variable was included in a separate logistic regression, controlling for age, gender, and K10 psychological distress score (as appropriate); AOR: Adjusted Odds Ratio - adjusted for the covariates; IOR: Interaction Odds Ratio, testing Predictor variable x Region interaction; bolded p-values are statistically significant (against Bonferroni-adjusted thresholds).
