# Supplementary material for: Determinants of suicidal ideation and suicide attempts: parallel cross-sectional analyses examining geographical location
Source: BMC Psychiatry. 2014 Jul 23;14:208. doi: 10.1186/1471-244X-14-208 (PMC4227072; doi:10.1186/1471-244X-14-208)
Supplement: Supplementary file 5 — Additional file 5: Selected logistic regressions for lifetime suicide attempts by remoteness for 20007-NSMHWB sample # . Selected logistic regressions for lifetime suicide attempts by remoteness for ARMHS sample#. (DOC 178 KB) [file 12888_2014_1706_MOESM5_ESM.doc]

**Supplementary Table S5a – Selected logistic regressions for lifetime suicide attempts by remoteness for 20007-NSMHWB sample^#^**

| **Characteristic**  **(predictor or exposure variable)** | **Category** | **Major cities (n=5388) n (%)** | **Inner Regional (n=1943) n (%)** | **Other (n=1132) n (%)** | **AOR** | **99% CI** | **p-value** | **IOR** | **p-value** |
| --- | --- | --- | --- | --- | --- | --- | --- | --- | --- |
| **Demographic factors** |  |  |  |  |  |  |  |  |  |
| Age in years | 18-44 | 118 (4.3) | 33 (4.2) | 25 (5.2) | . | . | . | . | . |
|  | 45-64 | 63 (4.1) | 28 (4.2) | 23 (6.4) | 1.1 | (0.78, 1.5) | 0.560 | 1.0 | 0.8293 |
|  | 65-85 | 12 (1.1) | 10 (2.0) | 5 (1.7) | **0.42** | **(0.22, 0.73)** | **<0.001** | 1.1 | 0.6493 |
| Gender | Male | 65 (2.7) | 24 (2.7) | 17 (3.3) | **.** | **.** | **.** | . | . |
|  | Female | 128 (4.3) | 47 (4.5) | 36 (5.8) | **1.4** | **(1.0, 2.0)** | **0.003** | 1.0 | 0.8460 |
| Marital status | Not married | 154 (5.3) | 56 (5.9) | 40 (6.6) | . | . | . | . | . |
|  | Married | 39 (1.6) | 15 (1.5) | 13 (2.5) | **0.36** | **(0.25, 0.52)** | **<0.001** | 1.0 | 0.8270 |
| Level of education | No university degree | 129 (4.0) | 54 (3.8) | 44 (5.0) | . | . | . | . | . |
|  | University or higher degree | 64 (3.0) | 17 (3.2) | 9 (3.5) | 0.69 | (0.49, 0.96) | 0.004 | 0.87 | 0.4728 |
| Employment Status | Employed | 119 (3.4) | 31 (2.7) | 26 (3.9) | . | . | . | . | . |
|  | Not in Workforce | 61 (3.4) | 37 (4.9) | 25 (5.9) | 1.5 | (1.1, 2.1) | 0.004 | 1.4 | 0.035 |
|  | Unemployed | 13 (11) | 3 (9.1) | 2 (5.6) | **2.3** | **(1.1, 4.5)** | **0.002** | 0.66 | 0.266 |
| Financial adversity | Low | 116 (2.5) | 44 (2.6) | 30 (3.2) | **.** | **.** | **.** | . | . |
|  | Medium | 49 (8.0) | 16 (7.1) | 18 (12) | **1.9** | **(1.3, 2.8)** | **<0.001** | 1.0 | 0.867 |
|  | High | 28 (24) | 11 (21) | 5 (19) | **4.3** | **(2.5, 7.3)** | **<0.001** | 0.78 | 0.360 |
| **Physical health** |  |  |  |  |  |  |  |  |  |
| Smoking | No | 104 (2.4) | 37 (2.5) | 22 (2.6) | **.** | **.** | **.** | . | . |
|  | Yes | 89 (8.0) | 34 (7.7) | 31 (11) | **2.7** | **(2.0, 3.8)** | **<0.001** | 1.2 | 0.315 |
| Number of chronic diseases | 0 | 127 (3.4) | 46 (3.9) | 34 (4.8) | . | . | . | . | . |
|  | 1 | 48 (4.0) | 17 (3.2) | 12 (4.1) | 1.3 | (0.80, 1.7) | 0.294 | 0.78 | 0.196 |
|  | >=2 | 18 (3.7) | 8 (3.4) | 7 (5.5) | 1.3 | (0.78, 2.4) | 0.160 | 0.96 | 0.885 |
| **Mental health** |  |  |  |  |  |  |  |  |  |
| Psychological distress (K10) | Low | 62 (1.6) | 23 (1.7) | 16 (1.9) | . | . | . | . | . |
|  | Moderate | 71 (5.8) | 28 (6.3) | 24 (9.6) | **3.7** | **(2.6, 5.2)** | **<0.001** | 1.2 | 0.338 |
|  | High | 60 (18) | 20 (17) | 13 (23) | **12** | **(7.9, 17)** | **<0.001** | 1.0 | 0.929 |
| Any affective disorder | No lifetime diagnosis | 72 (1.6) | 27 (1.6) | 23 (2.4) | **.** | **.** | **.** | . | . |
|  | Lifetime diagnosis with 12 month symptoms | 56 (17) | 20 (18) | 14 (21) | **5.1** | **(3.2, 7.9)** | **<0.001** | 0.92 | 0.693 |
|  | Lifetime diagnosis with no 12 month symptoms | 65 (13) | 24 (14) | 16 (15) | **6.4** | **(4.4, 9.2)** | **<0.001** | 0.89 | 0.519 |
| Any anxiety disorder | No lifetime diagnosis | 53 (1.3) | 18 (1.3) | 13 (1.6) | **.** | **.** | **.** | . | . |
|  | Lifetime diagnosis with 12 month symptoms | 96 (13) | 39 (15) | 30 (19) | **6.0** | **(4.0, 8.9)** | **<0.001** | 1.20 | 0.342 |
|  | Lifetime diagnosis with no 12 month symptoms | 44 (6.7) | 14 (5.5) | 10 (6.8) | **3.8** | **(2.4, 5.9)** | **<0.001** | 0.88 | 0.576 |
| Any substance use disorder | No lifetime diagnosis | 81 (1.9) | 35 (2.4) | 23 (2.9) | **.** | **.** | **.** | . | . |
|  | Lifetime diagnosis with 12 month symptoms | 35 (14) | 11 (14) | 7 (13) | **5.2** | **(3.2, 8.5)** | **<0.001** | 0.88 | 0.612 |
|  | Lifetime diagnosis with no 12 month symptoms | 77 (7.9) | 25 (6.1) | 23 (8.1) | **3.7** | **(2.6, 5.3)** | **<0.001** | 0.76 | 0.112 |
| Any lifetime psychiatric disorder | No | 12 (0.5) | 5 (0.6) | 5 (1.0) | **.** | **.** | **.** | . | . |
|  | Yes | 181 (6.6) | 66 (6.2) | 48 (7.7) | **7.7** | **(4.3, 14)** | **<0.001** | 0.74 | 0.286 |
| Any 12 month psychiatric disorder | No | 67 (1.6) | 26 (1.7) | 17 (1.9) | **.** | **.** | **.** | . | . |
|  | Yes | 126 (10) | 45 (10) | 36 (14) | **3.5** | **(2.4, 5.0)** | **<0.001** | 1.1 | 0.567 |
| Two or more psychiatric disorders | No | 39 (1.0) | 12 (0.8) | 8 (1.0) | **.** | **.** | **.** | . | . |
|  | Yes | 154 (11) | 59 (11) | 45 (14) | **8.1** | **(5.4, 12)** | **<0.001** | 1.2 | 0.368 |
| **Health Service Use** |  |  |  |  |  |  |  |  |  |
| Any professional mental health service use | No | 77 (1.7) | 23 (1.4) | 21 (2.1) | . | . | . | . | . |
|  | Yes | 116 (14) | 48 (18) | 32 (23) | **6.5** | **(4.6, 9.1)** | **<0.001** | 1.2 | 0.234 |
| Consulted a mental health professional in last 12 months and did not get as much help/info as needed | No | 46 (13) | 21 (20) | 12 (23) | . | . | . | . | . |
|  | Yes | 23 (27) | 4 (24) | 3 (25) | 1.4 | (0.70, 2.7) | 0.230 | 0.63 | 0.212 |

NSMHWB: National Survey of Mental Health and Well-being (aged 18-85).

# Bracketed values refer to the percentage of each predictor variable sub-category reporting lifetime suicide attempt; see supplementary Table S1 for cell sizes.

Note: Each predictor variable was included in a separate logistic regression, controlling for age, gender, and K10 psychological distress score (as appropriate); AOR: Adjusted Odds Ratio - adjusted for the covariates; IOR: Interaction Odds Ratio, testing Predictor variable x Region interaction; bolded p-values are statistically significant (against Bonferroni-adjusted thresholds).

**Supplementary Table S5b – Selected logistic regressions for lifetime suicide attempts by remoteness for ARMHS sample^#^**

| **Characteristic**  **(predictor or exposure variable)** | **Category** | **Inner Regional (n=251) n (%)** | **Other (n=383) n (%)** | **AOR** | **(99% CI)** | **p-value** | **IOR** | **p-value** |
| --- | --- | --- | --- | --- | --- | --- | --- | --- |
| **Demographic factors** |  |  |  |  |  |  |  |  |
| Age in years | 18-44 | 9 (15) | 9 (12) |  |  | . | . | . |
|  | 45-64 | 10 (7.4) | 15 (7.3) | 0.58 | (0.24, 1.4) | 0.103 | 0.82 | 0.766 |
|  | 65-85 | 1 (1.8) | 3 (3.0) | 0.21 | (0.05, 0.93) | 0.007 | 0.56 | 0.656 |
| Gender | Male | 10 (9.7) | 7 (4.8) |  |  | . | . | . |
|  | Female | 10 (6.8) | 20 (8.5) | 1.1 | (0.47, 2.6) | 0.759 | 0.36 | 0.128 |
| Marital status | Not married | 13 (14) | 19 (14) |  |  | . | . | . |
|  | Currently married | 7 (4.4) | 8 (3.3) | **0.29** | **(0.12, 0.69)** | **<0.001** | 1.4 | 0.611 |
| Level of education | No university degree | 15 (8.1) | 21 (6.6) |  |  | . | . | . |
|  | University or higher degree | 5 (7.6) | 6 (9.2) | 1.0 | (0.39, 2.7) | 0.956 | 0.81 | 0.785 |
| Employment Status | Employed | 5 (4.0) | 13 (6.3) |  |  | . | . | . |
|  | Not in Workforce | 11 (9.5) | 13 (8.1) | **3.0** | **(1.2, 7.7)** | **0.002** | 1.5 | 0.577 |
|  | Unemployed | 4 (44) | 1 (10) | 5.0 | (1.0, 24) | 0.009 | 7.9 | 0.146 |
| Financial adversity | Low | 5 (3.5) | 10 (4.5) |  |  | . | . | . |
|  | Medium | 8 (17) | 8 (11) | 3.0 | (1.1, 8.3) | 0.005 | 1.6 | 0.536 |
|  | High | 4 (22) | 5 (19) | 2.9 | (0.78, 11) | 0.036 | 1.4 | 0.741 |
| **Physical health** |  |  |  |  |  |  |  |  |
| Smoking | No | 11 (6.3) | 13 (5.0) |  |  | . | . | . |
|  | Yes | 7 (19) | 9 (15) | 2.1 | (0.79, 5.4) | 0.051 | 1.0 | 0.970 |
| Number of chronic diseases | 0 | 14 (9.9) | 19 (8.8) |  |  | . | . | . |
|  | 1 | 4 (4.6) | 5 (4.1) | 0.60 | (0.21, 1.7) | 0.218 | 0.79 | 0.770 |
|  | >=2 | 2 (8.7) | 3 (6.5) | 1.5 | (0.33, 6.6) | 0.502 | 1.4 | 0.770 |
| **Mental health** |  |  |  |  |  |  |  |  |
| Psychological distress (K10) | Low | 4 (5.7) | 3 (2.5) |  |  | . | . | . |
|  | Moderate | 6 (4.7) | 12 (6.3) | 1.3 | (0.41, 4.4) | 0.527 | 0.26 | 0.153 |
|  | High | 10 (19) | 12 (16) | **4.7** | **(1.4, 15)** | **<0.001** | 0.41 | 0.332 |
| Any affective disorder | No lifetime diagnosis | 6 (3.2) | 11 (3.6) |  |  | **.** | . | . |
|  | Lifetime diagnosis with 12 month symptoms | 11 (28) | 10 (26) | **6.7** | **(2.2, 20)** | **<0.001** | 1.3 | 0.742 |
|  | Lifetime diagnosis with no 12 month symptoms | 3 (12) | 6 (15) | **4.0** | **(1.3, 13)** | **0.002** | 0.81 | 0.817 |
| Any anxiety disorder | No lifetime diagnosis | 2 (1.5) | 7 (3.0) |  |  | **.** | . | . |
|  | Lifetime diagnosis with 12 month symptoms | 15 (19) | 14 (16) | **6.0** | **(2.0, 18)** | **<0.001** | 2.3 | 0.356 |
|  | Lifetime diagnosis with no 12 month symptoms | 3 (8.1) | 6 (9.5) | 3.6 | (1.0, 13) | 0.010 | 1.6 | 0.656 |
| Any substance use disorder | No lifetime diagnosis | 8 (4.1) | 13 (4.6) |  |  | . | . | . |
|  | Lifetime diagnosis with 12 month symptoms | 3 (38) | 3 (27) | **6.1** | **(1.3, 29)** | **0.003** | 1.8 | 0.614 |
|  | Lifetime diagnosis with no 12 month symptoms | 9 (18) | 11 (13) | **3.1** | **(1.2, 7.7)** | **0.001** | 2.1 | 0.295 |
| Any lifetime psychiatric disorder | No | 1 (1.1) |  |  |  | . | . | . |
|  | Yes | 19 (12) | 27 (11) | 19 | (1.4, 270) | 0.004 | 0.00 | 0.957 |
| Any 12 month psychiatric disorders | No | 3 (2.0) | 7 (2.9) |  |  | . | . | . |
|  | Yes | 17 (17) | 20 (14) | **5.0** | **(1.7, 14)** | **<0.001** | 2.0 | 0.396 |
| Two or more psychiatric disorders | No | 2 (1.4) | 7 (2.9) |  |  | **.** | . | . |
|  | Yes | 18 (17) | 20 (14) | **5.3** | **(1.8, 15)** | **<0.001** | 2.5 | 0.308 |
| **Health service use** |  |  |  |  |  |  |  |  |
| Any professional mental health service use | No | 8 (4.4) | 14 (5.0) |  |  | . | . | . |
|  | Yes | 12 (17) | 13 (13) | 2.5 | (1.0, 5.8) | 0.007 | 1.5 | 0.506 |
| Consulted a mental health professional in last 12months and did not get as much help/info as needed | No, needs met | 6 (13) | 7 (9.7) |  |  | . | . | . |
|  | Yes, needs not met | 8 (31) | 8 (22) | 2.1 | (0.68, 6.6) | 0.086 | 0.75 | 0.750 |

ARMHS: Australian Rural Mental Health Study, unweighted sample (aged 18-85) who completed the CIDI component.

# Bracketed values refer to the percentage of each predictor variable sub-category reporting lifetime suicide attempts; see supplementary Table S1 for cell sizes.

Note: Each predictor variable was included in a separate logistic regression, controlling for age, gender, and K10 psychological distress score (as appropriate); AOR: Adjusted Odds Ratio - adjusted for the covariates; IOR: Interaction Odds Ratio, testing Predictor variable x Region interaction; bolded p-values are statistically significant (against Bonferroni-adjusted thresholds).
